# The Metabolomic Profiling of the Flavonoid Compounds in Red Wine Grapes and the Impact of Training Systems in the Southern Subtropical Region of China

**DOI:** 10.3390/ijms25168624

**Published:** 2024-08-07

**Authors:** Huan Yu, Hong-Yan Li, Si-Hong Zhou, Guo Cheng, Rong-Fu Wei, Yong-Mei Zhou, Ying Zhang, Tai-Li Xie, Lan Zhang

**Affiliations:** 1 Grape and Wine Research Institute, Guangxi Academy of Agricultural Sciences, Nanning 530007, China; ankang1717@163.com (H.Y.); xiaoyan821025@163.com (H.-Y.L.); zhousihong@gxaas.net (S.-H.Z.); berry713@163.com (G.C.); rfw1819@126.com (R.-F.W.); zymzym78@163.com (Y.-M.Z.); zhangying@gxaas.net (Y.Z.); taili.xie@163.com (T.-L.X.); 2 Guangxi Key Laboratory of Fruits and Vegetables Storage-Processing Technology, Nanning 530007, China; 3 Agro-Food Science and Technology Research Institute, Guangxi Academy of Agricultural Sciences, 174 East Daxue Road, Nanning 530007, China

**Keywords:** flavonoids, wine grape varieties, winter fruits, training systems, southern subtropical region of China, metabolomics

## Abstract

Flavonoids play an important role in forming wine grapes and wine quality characteristics. The flavonoids of three winter red wine grapes, Yeniang No. 2 (YN2), Marselan (Mar), and Guipu No. 6 (GP6), were analyzed by ultra-high-performance liquid chromatography–triple quadrupole mass spectrometry (UPLC-QQQ-MS). Furthermore, the flavonoids in GP6 grapevines using two types of training systems, namely, trellis (T) and espaliers (E), were also compared in this study. Overall, 196 flavonoid metabolites, including 96 flavones, 38 flavonols, 19 flavanones, 18 polyphenols, 15 anthocyanins, 7 isoflavones, and 3 proanthocyanidins, were identified. The flavonoid profiles were remarkably different among these three grape varieties, while they did not change much in the GP6 managed on trellis and espaliers. Grape varieties with different genetic backgrounds have their own unique flavonoid profiles. Compared with Mar-T, isoflavones and flavonols presented higher contents in GP6-T and YN2-T, which mainly contain glycitein, genistin, calycosin, kaempferide, isotrifoliin, and ayanin. The anthocyanin content was significantly higher in YN2-T than in the other two varieties. YN2 and GP6-T present a more stable color, with significantly more acetylated diglucosides and methylated anthocyanins in YN2-T and GP6-T than in Mar-T. Notably, GP6 had more varied flavonoids and the better characteristics to its flavonoid profile out of these three varieties, due to it containing a higher number of anthocyanins, flavone, and flavonols and the greatest number of different flavonoid metabolites (DFMs), with higher contents than YN2 and Mar. Compared with the trellis training system, the espaliers training system increased the content of flavonoids detected in GP6 grape berries; however, the composition of flavonoids strictly depends on the grape variety.

## 1. Introduction

Flavonoids are the group of the most abundant biologically active phytonutrients among the polyphenolic compounds present in grapes, and they represent a large family of secondary metabolites, with nearly 6000 structures identified in plants [1]. Flavonoids are mainly divided into flavonols, flavan-3-ols, and anthocyanins [2]. Among grape berry secondary metabolites, flavonoids not only play a vital role in grapevine growth and development and stress responses but also contribute to the organoleptic qualities of grapes and wines [3,4,5]. Flavonols accumulate after flowering, during ripening, and in response to solar radiation, especially UV-B [6,7]. Flavan-3-ols are composed of a diversity of monomeric catechins and oligomeric or polymeric procyanidins, which are mainly responsible for wine’s astringency, bitterness, structure, and maturation [8]. Anthocyanins are pigments that contribute to the color of red grapes and wines, including cyanidin, delphinidin, malvidin, pelargonidin, peonidin, and petunidin, as well as further modifications such as glycosylation, methylation, and acylation [9,10]. Flavonoids are produced through the phenylpropanoid pathway, which is responsive to the environment [11]. Many factors have been identified that can influence flavonoids’ accumulation and composition in grapes, such as genotype [12,13,14], light, temperature [15,16], water status, soil type [17], plant growth regulators [18], and cultivation practices [19,20]. Training systems can regulate the microenvironment of the canopy by changing the temperature, humidity, and radiation for better spatial and light distribution and improve leaf photosynthetic efficiency to biosynthesize more flavonoids [20].

In general, training systems and other environmental factors can affect flavonoid content, but composition is mainly controlled by genotype [15,21,22]. Different *Vitis vinifera* L. red grape varieties can be distinguished by their flavonols, including some conjugates of quercetin, kaempferol, myricetin, and syringetin [23]. In grape skin, *Vitis labrusca* varieties were richer in catechin-gallate and epicatechin-gallate, while *V. vinifera* varieties accumulated higher levels of catechin, epicatechin, catechin-rhamnoside, and procyanidin B3 [24]. Quercetin was detected more in the skins of most *Vitis amurensis* cultivars and hybrids; however, more quercetin-3-*O*-rhamnoside was observed in non-*V. amurensis* East Asian species [13]. Furthermore, anthocyanins could be more useful for distinguishing grape varieties [25,26]. *V. vinifera* varieties generally contain only anthocyanin monoglucoside [27], but with the development of analytical technology, a small quantity of anthocyanin diglucosides have also been detected [10]. The concentration and composition of anthocyanins are different in wild Chinese grape species; malvidin-3,5-*O*-diglucoside, and coumaroyl derivatives are especially dominant [28].

Guangxi is located in the southern subtropical region of China, with high-temperature and high-humidity climate conditions. To fully utilize the characteristic climatic conditions in the southern region of China, rain shelter cultivation technology and a two-crop-a-year cultivation system were developed. Compared to summer grapes, the content of most flavonoids was higher in the winter grapes. Some grape varieties that can adapt to the local climate are often used as raw materials for wine processing. Marselan (*V. vinifera*, Mar) has great brewing prospects with its high flavanol content [29]. Yeniang No. 2 (*Vitis adenoclada*, YN2) currently is the wine variety with the largest planting area in Guangxi. In previous work, we found that YN2 was rich in phenolic acids and flavonols, but that its flavan-3-ol and anthocyanin contents were lower than those of *V. vinifera* varieties [30]. Guipu No. 6 (*Vitis* sp., GP6) is a good powdery mildew-tolerant variety collected in the field by the Guangxi Academy of Agricultural Sciences and Guangxi Academy of Specialty Crops in China [31]. Our previous research showed that GP6 berries are abundant in phenolic compounds [32]. In general, some studies on the flavonoids of East Asian species in southern China have been conducted [13,33,34,35]. Nevertheless, there is little information available on the detection of the flavonoids of the winter fruits of wine grapes with different training systems in the southern tropical region of China based on the high-throughput, highly sensitive, and wide-coverage analytical method of metabolomics.

In this study, a combination of ultra-high-performance liquid chromatography and triple quadrupole mass spectrometry was used to analyze and compare the flavonoid content of the winter fruits of red wine grapes grown in the southern subtropical region of China. The grape varieties tested were YN2, GP6, and Marselan (Mar). Furthermore, the flavonoid characteristics of GP6 berries that were, respectively, collected from grapevines using two types of training systems, namely, trellis (T) and espaliers (E), were also compared in this study. This study can provide a reference for the utilization of wine grape varieties in southern subtropical regions of China and the improvement of berry quality by cultivation treatment.

## 2. Results

### 2.1. Physical and Chemical Index Analysis

The comparison of physical and chemical indices across four treatments is shown in Table 1. The first, fresh weight per berry was remarkably different among the three varieties with the trellis training system. Specifically, GP6 had the highest berry weight among the three varieties, while Mar had the lowest berry weight. However, there were no significant differences in these indices between GP6-T and GP6-E.

### 2.2. Overview of Metabolic Profiling

**Figure 1:**
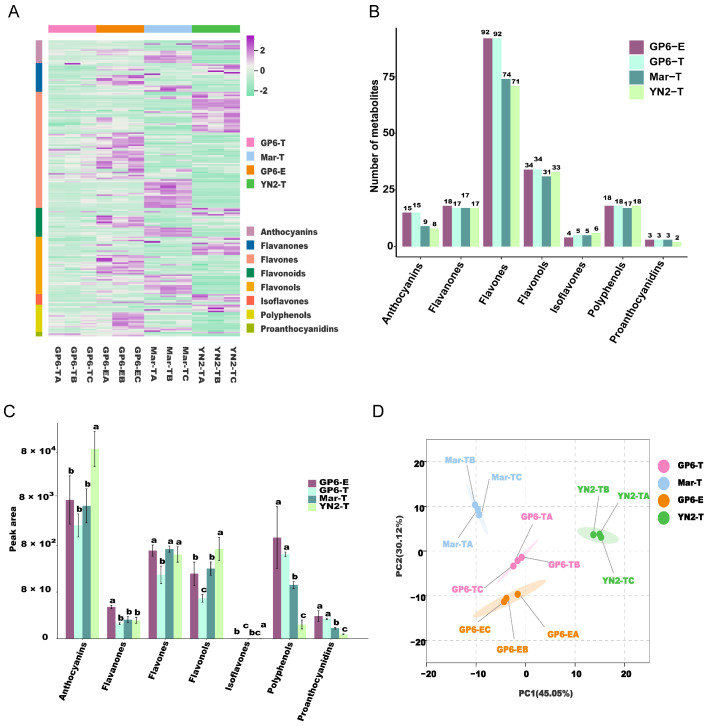
(**A**) Hierarchical cluster analysis (HCA). (**B**) The numbers of all groups of flavonoids in the four treatments. (**C**) The peak area of different groups of flavonoids. (**D**) Principal component analysis (PCA). Different letters indicate significant differences among samples according to Duncan’s test (*p* < 0.05).

Overall, 196 flavonoid metabolites, including 96 flavones, 38 flavonols, 19 flavanones, 18 polyphenols, 15 anthocyanins, 7 isoflavones, and 3 proanthocyanidins, were identified (Figure 1A, Table S1). The grape varieties differed significantly in their flavonoid metabolites (Figure 1A). YN2-T mainly contained flavones, flavonols, and polyphenols. Flavones, flavonols, and anthocyanins were the main components of Mar-T. The grapes from GP6-E were rich in flavones, flavonols, flavanones, and polyphenols. Of note, there were few differences in all flavonoids between GP6-E and GP6-T (Figure 1A). Compared with other flavonoid metabolites, the number of flavones was greatest in all four treatments. Additionally, the numbers of anthocyanins, flavones, and flavonols in GP6 were higher than those in the other varieties (Figure 1B). The number of isoflavones in YN2-T was highest among the three varieties. Based on the peak area of each metabolite, the flavonoids were identified as differentially accumulated among the four treatments (Figure 1C). The contents of anthocyanins and flavonols were significantly higher in YN2-T than in the other three samples. There were significantly more isoflavones in YN2-T and GP6-T. Polyphenols and proanthocyanidins accumulated at higher levels in the two samples of GP6. The numbers of different groups of flavonoids in GP6-E were totally identical to those in GP6-T, while their content was generally significantly higher than that in GP6-T. This indicated that the number of flavonoids mainly depended on the variety; however, the content of different groups of flavonoids was significantly increased by the espaliers training system compared to the trellis training system (Figure 1B,C).

In the PCA score plot (Figure 1D), YN2-T, Mar-T, GP6-T, and GP6-E were clearly separated, and the repeated samples were compactly gathered together. YN2-T was separated from Mar-T, GP6-T, and GP6-E by PC1, which explained 45.05% of the characteristics of the original dataset. Mar-T and YN2-T were distinguished from GP6-T and GP6-E by PC2, which explained 30.12% of the characteristics of the original dataset. This distinct separation indicated that varieties with different genetic backgrounds differed greatly in flavonoid composition and content.

**Figure 2:**
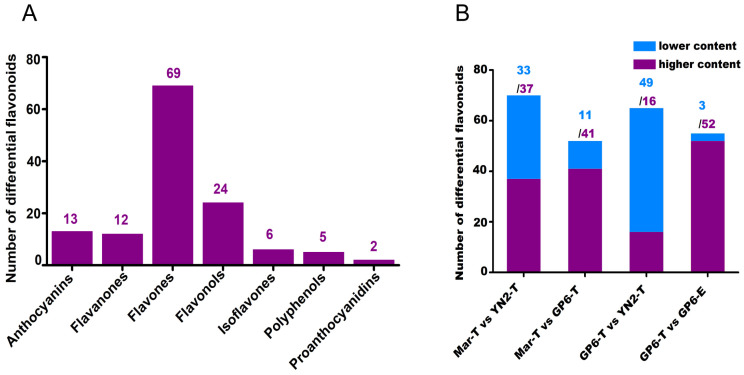
(**A**) Categorical metabolite statistics and (**B**) pairwise comparison of differential metabolites.

In order to clarify the different flavonoid metabolites (DFMs) among the four treatments, the numbers of different groups of flavonoids are shown in Figure 2A and the higher-content and lower-content metabolites of four pairwise comparisons are counted, as shown in Figure 2B. A total of 131 DFMs among the four treatments were observed, including 69 flavones, 24 flavonols, 13 anthocyanins, 12 flavanones, 6 isoflavones, 5 polyphenols, and 2 proanthocyanidins (Figure 2A). In Mar-T vs. YN2-T, Mar-T vs. GP6-T, GP6-T vs. YN2-T, and GP6-T vs. GP6-E were detected 70, 52, 65, and 55 DFMs, respectively (Figure 2B). In GP6-T and YN2-T, there were 37 and 52 DFMs that presented higher contents than those in Mar-T (Figure 2B). In GP6-T and YN2-T, there were 16 and 52 DFMs that presented higher contents than those in GP6-T, respectively (Figure 2B). The results above suggested that GP6 had the greatest number of DFMs with a higher content among the three varieties. On the other hand, most DFMs were higher in GP6 with espaliers training systems compared to trellis training systems.

### 2.3. Identification of Different Flavonoid Metabolites

**Figure 3:**
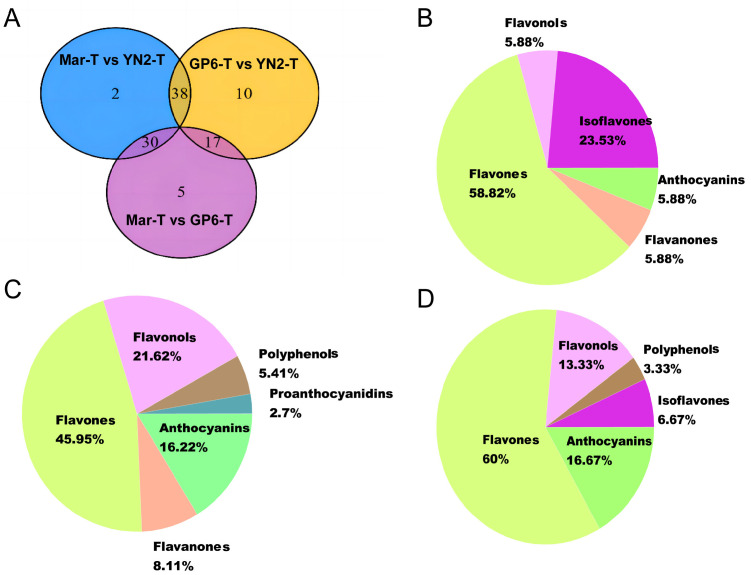
(**A**) Venn diagram illustrating the overlapping and specific differential metabolites of the three comparison groups (Mar-T vs. YN2-T, Mar-T vs. GP6-T, GP6-T vs. YN2-T). (**B**) The proportions of the 17 shared metabolites in GP6-T vs. YN2-T and Mar-T vs. GP6-T. (**C**) The proportions of the 38 shared metabolites in Mar-T vs. YN2-T and GP6-T vs. YN2-T. (**D**) The proportions of the 30 shared metabolites in Mar-T vs. YN2-T and Mar-T vs. GP6-T.

No overlap of DFMs was observed among the three grape variety comparison groups, indicating that grape varieties with different genetic backgrounds have their own unique flavonoid profile characteristics (Figure 3A). There were 10 unique differential metabolites between GP6-T and YN2-T, 5 between Mar-T and GP6-T, and 2 between Mar-T and YN2-T (Figure 3A).

For GP6-T vs. YN2-T and Mar-T vs. GP6-T, 17 common DFMs were considered to be the key metabolites distinguishing GP6-T from YN2-T and Mar-T (Figure 3A). The key metabolites were 58.82% flavones, 23.53% isoflavones, 5.88% flavanones, and 5.88% anthocyanins (Figure 3B). In addition, 38 DFMs were shared by Mar-T vs. YN2-T and GP6-T vs. YN2-T, and the proportion of each class was 45.95% flavones, 21.62% flavonols, 16.22% anthocyanins, 8.11% flavanones, 5.41% polyphenols, and 2.70% proanthocyanins (Figure 3C). Thirty common DFMs were regarded as the major contributors to the variety variation between Mar-T vs. YN2-T and Mar-T vs. GP6-T, including 60% flavones, 13.33% flavonols, 16.67% anthocyanins, 6.67% isoflavones, and 3.33% polyphenols (Figure 3D).

### 2.4. KEGG Annotation and Enrichment Analysis of Differential Metabolites

**Figure 4:**
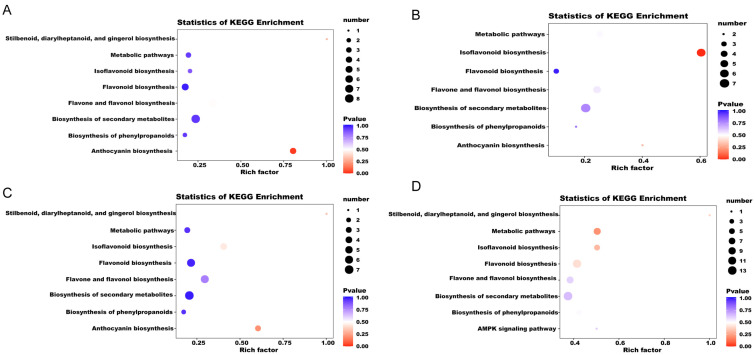
KEGG pathway analysis of differential metabolites in four comparison groups: (**A**) Mar-T vs. YN2-T, (**B**) Mar-T vs. GP6-T, (**C**) GP6-T vs. YN2-T, and (**D**) GP6-T vs. GP6-E.

The differential metabolites of each comparison group were annotated using the Kyoto Encyclopedia of Genes and Genomes (KEGG) database. The above-mentioned annotated results were classified and enriched based on the pathway types in KEGG, and the enrichment results of each comparison group are shown in Figure 4. Based on the enrichment results, we observed that the DFMs of the comparison groups were mainly distributed in pathways including anthocyanin biosynthesis; the biosynthesis of phenylpropanoids; the biosynthesis of secondary metabolites; flavone and flavonol biosynthesis; flavonoid biosynthesis; stilbenoid, diarylheptanoid, and gingerol biosynthesis; isoflavonoid biosynthesis; and metabolic pathways (Figure 4). The flavonoid metabolites that caused the differences between YN2-T and GP6-T and Mar-T were significantly (*p* value < 0.05) enriched in anthocyanin biosynthesis (Figure 4A,C). When comparing Mar-T and GP6-T, the significantly enriched metabolic pathways were related to isoflavonoid biosynthesis (*p* value < 0.05) (Figure 4B). Metabolic pathways related to isoflavonoid biosynthesis and flavonoid biosynthesis were significantly enriched (*p* value < 0.05) when comparing GP6-T vs. GP6-E (Figure 4D).

### 2.5. Differential Metabolites of Flavonoid Biosynthesis Pathways

**Figure 5:**
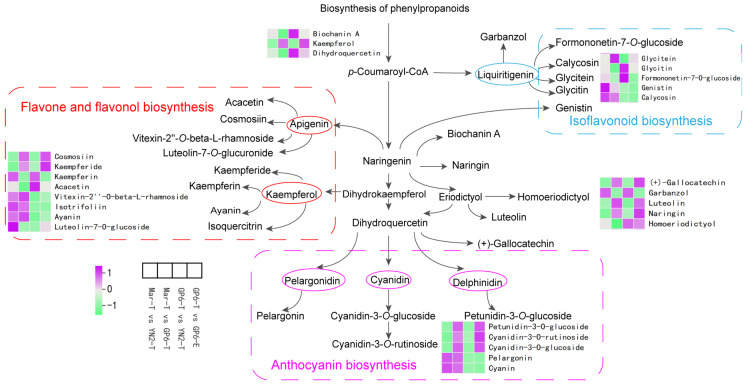
Flavonoid biosynthesis pathways in the pairwise comparisons of four treatments. The log_2_-transformed FPKM values were used to prepare the heatmaps and present the biological pathways connected with different metabolite expression levels.

Based on the KEGG database, a flavonoid biosynthesis network containing 26 DFMs was constructed, with approximately three branches from p-coumaroyl-CoA (Figure 5).

For flavone and flavonol biosynthesis, in YN2-T, certain compounds were found in higher contents than in Mar-T, mainly kaempferin, vitexin-2″-O-beta-L-rhamnoside, isotrifoliin, ayanin, and luteolin-7-O-glucoside. GP6-T had a higher content of some compounds than that of Mar-T, mainly cosmosiin, kaempferide, vitexin-2″-O-beta-L-rhamnoside, isotrifoliin, and ayanin. In GP6-T, the content of some compounds was higher than in YN2, mainly cosmosiin, vitexin-2″-O-beta-L-rhamnoside, isotrifoliin, ayanin, and luteolin-7-O-glucoside. Compared with GP6-T, higher contents of cosmosiin and kaempferide were detected in GP6-E, while lower contents of kaempferin, acacetin, vitexin-2″-O-beta-L-rhamnoside, isotrifoliin, ayanin, and luteolin-7-O-glucoside were detected.

For isoflavonoid biosynthesis, in Mar-T, some compounds were found in lower contents than in YN2-T or GP6-T, mainly glycitein, genistin, and calycosin. In YN2-T, the content of some compounds, including glycitein, genistin, and calycosin, was lower than in GP6-T. Compared with GP6-T, lower contents of formononetin-7-O-glucoside, genistin, and calycosin were detected in GP6-E.

For anthocyanin biosynthesis, in YN2-T, the content of some compounds was higher than in Mar-T, mainly pelargonin and cyanin. In GP6-T, the content of some compounds was higher than in Mar-T, mainly petunidin-3-O-glucoside, cyanidin-3-O-rutinoside, cyanidin-3-O-glucoside, pelargonin, and cyanin. In addition, the content of these compounds in YN2-T was lower than that in GP6-T. Compared with GP6-T, higher contents of petunidin-3-O-glucoside, cyanidin-3-O-rutinoside, and cyanidin-3-O-glucoside were detected in GP6-E, while lower contents of pelargonin and cyanin were detected in GP6-E.

### 2.6. Differentially Modified Anthocyanins

**Figure 6:**
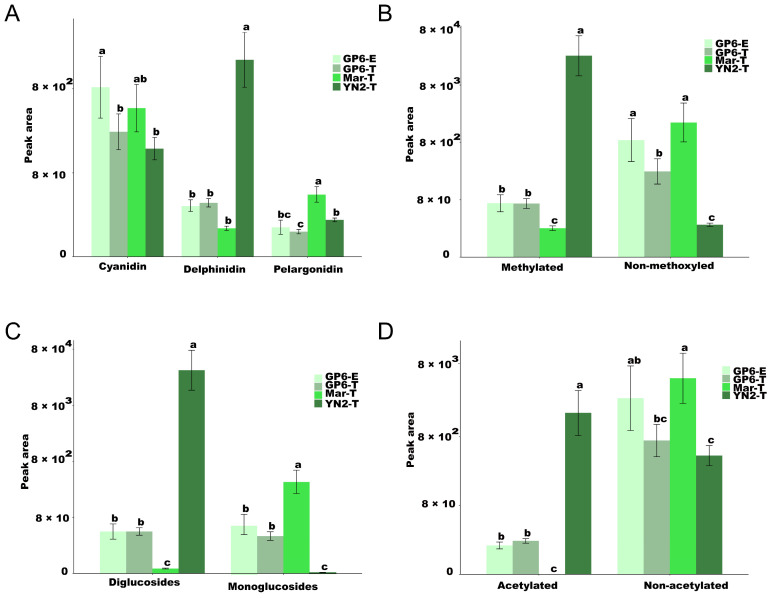
(**A**) Cyanidin, Delphinidin and Pelargonidin modified anthocyanins, (**B**) Methylated and Non-methoxyled modified anthocyanins, (**C**) Diglucosides and Monoglucoside modified anthocyanins, (**D**) Acetylated and Non-acetylated modified anthocyanins. The peak area of different modified anthocyanins among the four treatments. Different letters indicate significant differences among samples according to Duncan’s test (*p* < 0.05).

To investigate modifications, the differences in anthocyanidin among the four treatments are displayed in Figure 6 and Table S2. The anthocyanins’ composition and content varied significantly among varieties (Figure 6). The content of some different modified anthocyanins was significantly higher in YN2-T than in the other varieties, including delphinidin, acetylated diglucosides, and methylated anthocyanins. GP6-T was quite different from Mar-T, which was characterized by higher contents of acetylated diglucosides and methylated anthocyanins. However, compared to the other varieties, Mar-T has been demonstrated to be rich in pelargonidin, non-methoxyled monoglucoside, and non-acetylated derivatives in its berry skin, without any acetylated anthocyanins being detected (Figure 6).

In the case of calculating anthocyanin modifications, the number of different modified anthocyanins detected in GP6-T was greater than that in other varieties (Table S2). Large quantities of cyanidin were detected in the four treatments, followed by delphinidin and pelargonidin. Compared with methylated anthocyanins, greater amounts of non-methoxyled anthocyanin derivatives were detected in the four treatments. The number of diglucosides and monoglucosides showed no difference in GP6-T and GP6-E, while more diglucosides were detected in YN2-T and fewer diglucosides were found in Mar-T. In terms of the number of different modified anthocyanins, there was no difference between GP6-T and GP6-E, while their quantities were generally different, especially those of cyanidin and non-methoxyled anthocyanins, which were significantly higher in GP6 with espalier training systems compared to with trellis training systems (Duncan test, *p* < 0.05), indicating that the types and number of different modified anthocyanins mainly depend on varieties; however, their content depends on training systems (Table S2).

## 3. Discussion

Grapevine (*V. vinifera*) is popular for its abundant polyphenolic compounds, especially flavonoids, which are beneficial for fruit quality and defense against damage. In a previous study, we observed a total of 774 metabolites via widely targeted metabolomics detection in two wine grape varieties; in particular, 157 types of flavonoids were detected in 400 secondary metabolites, while the invasion of E. necator stimulated flavonoid increases [31]. In this study, we characterized the physicochemical indices and flavonoid differences in four grape samples. A w metabolite profiling analysis identified 196 flavonoids, including 96 flavones, 38 flavonols, 19 flavanones, 18 polyphenols, 15 anthocyanins, 7 isoflavones, and 3 proanthocyanidins. The accumulation of flavonoid metabolites has been proven to differ depending on species [2,14,21]. The flavonoid metabolic network we proposed here was based on the differential flavonoid metabolites, which revealed that the three varieties were clearly distinguished by their flavonoid profiles. Genotypes strictly regulate the content and composition of flavonoids [14], while climatic conditions, agricultural practices, and other biotic factors also modulate flavonoid profiles, especially training systems [15,16,19,20]. In this study, espalier training systems increased the content of flavonoids compared to trellis training systems.

The composition and content of flavonoids vary greatly with diverse genetic backgrounds. Zhu, L. et al. [13] found that the total content and composition of each flavonoid type varied significantly among different grape varieties, including European, East Asian, and North American varieties and Euro-Asian hybrids and Euro-American hybrids, as well as two varieties of muscadine grapes. Compared with populations located in southern Spain, richer anthocyanins and poorer flavonols were collected in wild grapevine populations located in northern Spain [36]. In the present study, there were no common DFMs observed among the test grape varieties, indicating that grape varieties with different genetic backgrounds have their own unique flavonoid profile characteristics. The number and content of anthocyanins, flavones, flavonols, polyphenols, and proanthocyanidins differed among the test varieties. The anthocyanin content was significantly higher in YN2-T than in the other two varieties, as pointed out previously [37,38]. However, the anthocyanin content in YN2 was lower than that in V. vinifera varieties [30]. This difference may be explained by anthocyanins being affected by ecological conditions, viticulture practices, and other factors [10]. Isoflavones and flavonols also play an important role in the cross-talk between plants and microorganisms [7,39]. Compared with Mar-T, isoflavones and flavonols were present in higher contents in GP6-T and YN2-T, which mainly contain luteolin-7-O-glucoside, ayanin, isotrifoliin, and vitexin-2″-O-beta-L-rhamnoside. These data suggested that GP6-T and YN2-T have better disease resistance than Mar-T [31]. Anthocyanins are secondary metabolites, while water-soluble pigments belonging to the flavonoids, acetylated diglucosides, and methylated anthocyanins groups are more stable structures [38,40]. Higher contents of pelargonin and cyanin were detected in GP6-T and YN2-T than in Mar-T. YN2 was dominated by anthocyanidin-3,5-O-diglucosides. The content of anthocyanins with more stable structures, including acetylated diglucosides and methylated anthocyanins, was significantly higher in YN2-T and GP6-T. All these were quite different from Mar-T, which was characterized by pelargonidin, non-methoxyled monoglucoside, and non-acetylated derivatives in its berry skin, without any acetylation being detected. It can be concluded that YN2-T and GP6-T present a more stable color [38,41]. Astringency, sweetness, bitterness, salivary viscosity, sourness, aroma, and color formation can be imparted by proanthocyanidins [42]. In the present study, proanthocyanidins and polyphenols accumulated at higher levels in two samples of GP6, which suggested that the skin of GP6 berries showed better sensory characteristics for wine. As can be noted, the number of anthocyanins, flavone, and flavonols in GP6 was higher than that in other varieties, indicating that GP6 had more varied flavonoids. Additionally, we found that GP6 had the greatest number of DFMs, with a higher content than YN2 and Mar, indicating that GP6 showed the better characteristics in its flavonoid profile of those three varieties. Combined with previous research findings [32], we can conclude that GP6 berries are excellent sources of flavonoids, which leads to a high potential for wine in the South China region.

The types of training systems used may lead to differences in total leaf area and the percentage of the leaf area well exposed to light [43]. Consequently, the training system and the accompanying light microclimate of leaves play an important role in the ability of a grapevine to photosynthesize efficiently [43]. There are many studies available reporting the influence of training systems on berry composition, including soluble solids, phenolic compounds, anthocyanin content, flavonols, and volatile compounds [20,44,45,46]. To choose an appropriate training system for increasing the flavonoids in GP6, the espalier training system was compared with the trellis training system from a flavonoid point of view. The results of this work indicate that the quantity of different groups of flavonoids was similar between the two training systems, which again certified that variety had a strict regulation of the composition of flavonoids. Meanwhile, the content of flavonoids was generally significantly higher in GP6-E than in GP6-T. Compared with the trellis training system, this result may be related to the horizontal growth of espalier branches, as there is a certain angle between the mother branch and the fruiting branch which weakens the advantage of the top of the new shoot and is conducive to transporting more nutrients to the fruit [47]. On the other hand, the horizontal leaf curtain of espalier training systems significantly reduced the temperature and humidity fluctuation range and the high temperature ratio of the fruit surrounding the environment, resulting in an increased content of flavonoids in the skins [47]. In conclusion, the content of flavonoids varied between different training systems, and espalier training systems help to increase the content of flavonoids in grape berries compared to trellis training systems; however, the composition of flavonoids strictly depends on the variety grown.

## 4. Materials and Methods

### 4.1. Experimental Vineyard Conditions

We conducted this experiment in 2018 in the vineyards of the Grape and Wine Research Institute, Guangxi Academy of Agricultural Sciences, located in Nanning, Guangxi Province, China (22°36′39″ N, 108°13′51″ E). We experimented with three grape varieties: YN2, Mar, and GP6. For this study, the trellis system was applied to YN2 and Mar (YN2-T, Mar-T), while both the trellis training system and espalier training system were used on GP6 (GP6-T, GP6-E). The trellis training system and espalier training system are shown in Figure S1. The daily management of the vineyard, including pest and water control methods, was carried out according to previous standards [31]. These plants were grown in four treatments for this study: YN2-T, Mar-T, GP6-T, and GP6-E. Each treatment had three biological replicates, and every treatment comprised more than five plants.

### 4.2. Berry Sampling and Physical Chemical Index Analysis

Each biological replicate contained more than 100 berries that were randomly sampled at harvest time (E-L 38). Besides the winter fruits of YN2, which were harvested in early November, other varieties’ winter fruits were harvested in the first half of December. Physical and chemical measurements were conducted on more than 20 berries from each biological replicate after transport to the laboratory, including berry fresh weight, total soluble solids (TSSs) content, and titratable acidity (TA). The TSS assessment was carried out using a refractometer (Digital Hand-held Pocket Refractometer PAL-1, Atago, Tokyo, Japan). The TA was determined as tartaric acid equivalents via acid–alkali titration with NaOH at pH 8.2. The skin of the remaining berries was collected and immediately frozen in liquid nitrogen for metabolomics analysis.

### 4.3. Sample Preparation and Metabolite Extraction

A mixer mill (MM 400, Retsch, Shanghai, China) with a zirconia bead was used to crush freeze-dried berry skin for 1.5 min at 30 Hz. One hundred milligrams of powder was weighed and extracted overnight at 4 °C with 1.0 mL of 70% aqueous methanol. After centrifugation at 10,000 g for 10 min, the extracts were absorbed (CNWBOND Carbon–GCB SPE Cartridge, 250 mg, 3 mL; ANPEL, Shanghai, China, www.anpel.com.cn/cnw, accessed on 26 January 2019) and filtered (SCAA-104, 0.22 μm pore size; ANPEL, Shanghai, China, http://www.anpel.com.cn/, accessed on 26 January 2019) before LC–MS analysis.

### 4.4. HPLC Conditions

The sample extracts were analyzed using an LC–ESI–MS/MS system (HPLC, Shim-pack UFLC SHIMADZU CBM30A system, www.shimadzu.com.cn/, accessed on 26 January 2019; MS, Applied Biosystems 6500 Q TRAP, www.appliedbiosystems.com, accessed on 26 January 2019). The analytical conditions were set according to previous reports [48]. The effluent was alternatively connected to an ESI-triple quadrupole-linear ion trap (Q TRAP)–MS.

### 4.5. ESI-Q TRAP-MS/MS

LIT and triple quadrupole (QQQ) scans were acquired on a triple quadrupole-linear ion trap mass spectrometer (Q TRAP), API 6500 Q TRAP LC/MS/MS System, equipped with an ESI Turbo Ion-Spray interface, operating in a positive ion mode and controlled by Analyst 1.6.3 software (AB Sciex). The ESI source operation parameters were consistent with the literature [48]. A specific set of MRM transitions was monitored for each period according to the metabolites eluted within this period.

### 4.6. Principal Component Analysis (PCA) and Hierarchical Cluster Analysis (HCA)

A PCA was performed to preliminarily understand the overall metabolic difference between the 12 grape samples using the statistics function prcomp within R (www.r-project.org, accessed on 29 January 2019). The data were unit variance-scaled before an unsupervised PCA. After analyzing the HCA, the results were shown in the form of heatmaps with dendrograms. We carried out the PCA and HCA using the R package heatmap.

### 4.7. Differential Metabolite Analysis

The significantly differential metabolites obtained by pairwise comparison were screened using the following conditions: VIP ≥ 1 and absolute log_2_FC (fold change) ≥ 1. The VIP values were extracted from the OPLS-DA results, which also contained score plots and permutation plots generated using the R package MetaboAnalystR. The data were log-transformed (log_2_) and mean-centered before OPLS-DA. To avoid overfitting, a permutation test (200 permutations) was performed.

### 4.8. Kyoto Encyclopedia of Genes and Genomes (KEGG) Annotation and Enrichment Analysis

Based on the KEGG Compound database (http://www.kegg.jp/kegg/compound/, accessed on 29 January 2019), we identified the metabolites. On the basis of the *p*-values of the hypergeographic test, we calculated the significance of pathways with significant regulatory metabolites in an MSEA (metabolite set enrichment analysis).

## 5. Conclusions

In this study, the composition and content of flavonoid-related metabolites were surveyed by ultra-high-performance liquid chromatography–triple quadrupole mass spectrometry (UPLC-QQQ-MS)-based metabolomics using two comparisons: one comparison was three red wine grapes grown with the same training systems, and the other was the same variety grown with two training systems. A total of 196 flavonoids, including 96 flavones, 38 flavonols, 19 flavanones, 18 polyphenols, 15 anthocyanins, 7 isoflavones, and 3 proanthocyanidins, were observed. Flavonoid content largely varied with different genetic backgrounds. GP6 and YN2 showed better characteristics in their flavonoid profiles than Mar, especially GP6, which is a potential resource, with good brewing quality characteristics, for the South China region. Compared with the trellis training system, the espalier training system increased the content of flavonoids in the grape berries; however, the composition of flavonoids strictly depended on the variety. So, espalier training systems are recommended for growing GP6 with a higher content of flavonoids in the South China region. To better understand the effects of trellis training and espalier training on flavonoids, these two training systems will be applied to GP6, YN2, and Mar for three years.

## Figures and Tables

**Table 1:** Physical and chemical index analysis.

Physical and Chemical Index	YN2-T	Mar-T	GP6-T	GP6-E
Berry weight/(g)	0.97 ± 0.13 b	0.73 ± 0.06 c	1.83 ± 0.06 a	1.73 ± 0.19 a
Soluble solids concentration/(°Brix)	16.53 ± 0.85 a	16.2 ± 0.35 a	17.27 ± 0.15 a	16.9 ± 1.71 a
Titratable acidity/(g·L^−1^)	14.34 ± 3.55 a	19.29 ± 0.99 a	18.27 ± 1.48 a	18.55 ± 3.06 a

Note: Data represent the mean ± standard deviation (*n* = 3). In each row, different letters indicate significant differences among samples according to Duncan’s test (*p* < 0.05).

## Data Availability

The data used for the analysis in this study are available within the article and the Supplementary Materials.

## Supplementary Materials

The following supporting information can be downloaded at https://www.mdpi.com/article/10.3390/ijms25168624/s1.
