# Supplementary material for: The Metabolomic Profiling of the Flavonoid Compounds in Red Wine Grapes and the Impact of Training Systems in the Southern Subtropical Region of China
Source: Int J Mol Sci. 2024 Aug 7;25(16):8624. doi: 10.3390/ijms25168624 (PMC11354489; doi:10.3390/ijms25168624)
Supplement: Supplementary file 1 [file ijms-25-08624-s001.zip › Table S1.pdf]

Table S1 The information of all detected metabolites

Index	Q1 (Da)	Q3 (Da)	Rt (min)	Molecular Weight (Da)	Ionization model	KEGG ID	Compounds	Class	mix01	mix02	mix03	GP6-TA	GP6-TB	GP6-TC	GP6-EA	GP6-EB	GP6-EC	Mar-TA	Mar-TB	Mar-TC	YN2-TA	YN2-TB	YN2-TC
pma0724	4.35E+02	2.85E+02	3.79E+00	4.34E+02	[M+H]+	-	Naringenin-C-hexoside	Flavone	6.48E+04	6.65E+04	4.67E+04	7.59E+04	1.11E+05	7.94E+04	1.71E+05	1.26E+05	1.85E+05	9.00E+00	9.00E+00	9.00E+00	1.12E+05	7.60E+04	8.45E+04
pma0757	5.65E+02	3.31E+02	4.27E+00	5.64E+02	[M+H]+	-	Tricin-O-oxalylhexoside	Flavone	5.01E+05	6.20E+05	4.20E+05	8.38E+05	7.53E+05	9.22E+05	6.01E+05	9.58E+05	8.02E+05	4.23E+05	4.81E+05	4.37E+05	9.00E+00	9.00E+00	9.00E+00
pma0760	5.65E+02	3.17E+02	4.33E+00	5.64E+02	[M+H]+	-	Selgin-O-malonylhexoside	Flavone	2.92E+05	3.75E+05	3.09E+05	1.83E+05	1.80E+05	1.45E+05	8.14E+05	5.62E+05	3.73E+05	5.19E+05	4.81E+05	5.69E+05	9.00E+00	9.00E+00	9.00E+00
pma0779	7.25E+02	3.31E+02	4.50E+00	7.24E+02	[M+H]+	-	Tricin-O-rhamnosyl-O-malonylhexoside	Flavone	1.30E+05	1.45E+05	1.27E+05	5.33E+04	6.54E+04	1.16E+05	6.06E+04	1.02E+05	9.64E+04	9.00E+00	9.00E+00	9.00E+00	4.44E+05	3.24E+05	4.76E+05
pma0791	5.21E+02	2.73E+02	4.50E+00	5.20E+02	[M+H]+	-	Naringenin-O-malonylhexoside	Flavanone	5.19E+05	7.41E+05	4.63E+05	7.53E+05	8.18E+05	1.15E+06	9.79E+05	1.37E+06	1.16E+06	5.43E+05	6.23E+05	5.26E+05	9.00E+00	9.00E+00	9.00E+00
pma0819	7.29E+02	3.31E+02	5.19E+00	7.28E+02	[M+H]+	-	Tricin-O-glyceryl-hexosyl-O-hexoside	Flavone	1.09E+05	1.01E+05	1.21E+05	5.05E+04	6.14E+04	6.82E+04	4.05E+04	5.49E+04	5.45E+04	2.46E+05	2.59E+05	1.71E+05	9.00E+00	9.00E+00	9.00E+00
pma0825	5.03E+02	2.55E+02	5.24E+00	5.02E+02	[M+H]+	-	Chrysin-O-malonylhexoside	Flavone	2.01E+04	3.03E+04	2.98E+04	3.18E+04	2.85E+04	4.70E+04	4.39E+04	7.00E+04	3.42E+04	9.00E+00	9.00E+00	9.00E+00	9.00E+00	9.00E+00	9.00E+00
pma1108	4.33E+02	2.83E+02	3.78E+00	4.32E+02	[M+H]+	-	Apigenin-C-glucoside	Flavone	4.81E+04	5.31E+04	3.51E+04	3.21E+04	4.68E+04	3.50E+04	4.83E+04	7.92E+04	8.63E+04	9.00E+00	9.00E+00	6.33E+04	9.00E+00	9.00E+00	2.10E+04
pma1116	3.01E+02	2.86E+02	4.13E+00	3.00E+02	[M+H]+	C10098	Kaempferide	Flavonol	6.28E+06	6.52E+06	7.01E+06	4.88E+06	3.33E+06	5.75E+06	6.61E+06	7.39E+06	5.19E+06	1.50E+07	1.64E+07	1.44E+07	9.00E+00	1.56E+05	1.24E+05
pma1590	4.63E+02	3.01E+02	2.89E+00	4.63E+02	Protonated	-	Peonidin-O-hexoside	Anthocyanins	1.10E+07	1.20E+07	1.06E+07	1.13E+07	9.89E+06	1.13E+07	1.41E+07	1.36E+07	1.09E+07	1.96E+07	2.04E+07	1.74E+07	9.00E+00	9.00E+00	9.00E+00
pma6199	4.17E+02	2.55E+02	4.99E+00	4.16E+02	[M+H]+	-	Chrysin-O-hexoside	Flavone	3.40E+05	5.00E+05	3.72E+05	9.13E+04	9.66E+04	1.86E+05	2.87E+05	2.40E+05	2.02E+05	1.13E+06	1.82E+06	1.07E+06	9.14E+04	6.93E+04	9.92E+04
pma6360	4.77E+02	2.97E+02	4.06E+00	4.76E+02	[M+H]+	-	O-methylChrysoeriol-8-C-hexoside	Flavone	5.62E+04	3.96E+04	5.08E+04	9.00E+00	9.00E+00	2.15E+04	9.00E+00	1.88E+04	9.00E+00	1.94E+04	9.00E+00	2.74E+04	1.60E+05	1.11E+05	1.67E+05
pma6389	3.45E+02	1.77E+02	6.33E+00	3.44E+02	[M+H]+	C04444	Ayanin	Flavonol	1.02E+04	1.57E+04	1.28E+04	8.63E+03	1.39E+04	1.93E+04	3.26E+04	3.72E+04	3.07E+04	9.00E+00	9.00E+00	9.00E+00	4.51E+03	6.19E+03	4.26E+03
pma6499	5.09E+02	3.47E+02	3.41E+00	5.08E+02	[M+H]+	-	Limocitrin-O-hexoside	Flavone	5.27E+06	5.82E+06	5.78E+06	4.84E+06	5.42E+06	7.54E+06	1.13E+07	4.96E+06	5.59E+06	9.00E+00	9.00E+00	9.00E+00	2.73E+07	1.80E+07	2.80E+07
pma6516	7.57E+02	5.95E+02	3.55E+00	7.56E+02	[M+H]+	-	C-hexosyl-apigenin-O-hexosyl-O-hexoside	Flavone	8.70E+05	9.84E+05	9.01E+05	1.60E+05	1.85E+05	2.70E+05	5.18E+05	3.98E+05	2.77E+05	9.00E+00	9.00E+00	9.00E+00	5.91E+06	5.45E+06	5.23E+06
pma6576	3.47E+02	1.53E+02	4.41E+00	3.46E+02	[M+H]+	-	Spinacetin	Flavone	9.20E+05	1.15E+06	8.75E+05	1.24E+06	1.14E+06	1.25E+06	1.76E+06	1.26E+06	8.66E+05	7.24E+05	7.16E+05	6.92E+05	1.62E+06	1.39E+06	1.62E+06
pma6639	4.79E+02	3.17E+02	4.17E+00	4.78E+02	[M+H]+	-	Isorhamnetin-O-hexoside	Flavonol	4.40E+06	6.31E+06	4.61E+06	4.21E+06	3.70E+06	3.86E+06	1.92E+07	1.03E+07	1.03E+07	2.72E+06	2.88E+06	3.54E+06	5.58E+06	3.39E+06	5.03E+06
pma6687	5.57E+02	3.31E+02	4.44E+00	5.56E+02	[M+H]+	-	Tricin-4'-O-(syringyl-glyceryl)ether	Flavone	3.37E+05	3.88E+05	2.44E+05	7.72E+04	7.09E+04	1.25E+05	7.07E+04	1.22E+05	5.43E+04	5.59E+05	6.15E+05	5.23E+05	9.00E+00	9.00E+00	9.00E+00
pmb0322	6.53E+02	3.45E+02	4.76E+00	6.52E+02	[M+H]+	-	"3',4',5'-Tricetin-O-rutinoside"	Flavone	6.78E+04	1.06E+05	6.96E+04	6.50E+04	4.80E+04	6.07E+04	4.87E+04	5.60E+04	4.97E+04	1.00E+05	1.61E+05	1.64E+05	9.00E+00	9.00E+00	9.00E+00
pmb0541	6.97E+02	5.35E+02	2.09E+00	6.97E+02	Protonated	-	Cyanidin-3-O-glucosyl-malonylglucoside	Anthocyanins	3.30E+06	3.22E+06	2.96E+06	9.15E+05	8.78E+05	1.06E+06	1.54E+06	5.95E+05	7.97E+05	9.00E+00	9.00E+00	9.00E+00	3.61E+06	3.85E+06	3.72E+06
pmb0545	4.77E+02	3.15E+02	3.32E+00	4.77E+02	Protonated	-	Rosinidin-O-hexoside	Anthocyanins	7.35E+05	8.53E+05	7.88E+05	1.42E+06	1.23E+06	1.88E+06	1.80E+06	2.10E+06	1.88E+06	9.00E+00	9.00E+00	9.00E+00	9.00E+00	9.00E+00	9.00E+00
pmb0563	3.01E+02	2.86E+02	3.95E+00	3.01E+02	Protonated	C08726	Peonidin	Anthocyanins	2.85E+06	3.41E+06	3.06E+06	1.74E+06	1.62E+06	2.22E+06	2.44E+06	2.79E+06	1.84E+06	7.37E+06	6.14E+06	6.51E+06	9.00E+00	9.00E+00	9.00E+00
pmb0565	5.09E+02	3.47E+02	3.41E+00	5.08E+02	[M+H]+	-	Syringetin-3-O-hexoside	Flavonol	5.46E+06	6.38E+06	5.78E+06	5.53E+06	5.64E+06	8.46E+06	1.10E+07	5.44E+06	5.35E+06	9.00E+00	9.00E+00	9.00E+00	2.55E+07	1.83E+07	2.80E+07
pmb0569	5.09E+02	3.47E+02	4.17E+00	5.08E+02	[M+H]+	-	Syringetin-5-O-hexoside	Flavone	5.62E+06	6.99E+06	5.87E+06	5.77E+06	5.51E+06	7.09E+06	1.13E+07	8.28E+06	6.84E+06	4.37E+06	4.40E+06	4.62E+06	9.66E+06	9.25E+06	1.15E+07
pmb0580	4.17E+02	2.55E+02	5.00E+00	4.16E+02	[M+H]+	-	Chrysin-5-O-glucoside-(Toringin)	Flavone	2.95E+05	5.05E+05	3.83E+05	6.51E+04	9.86E+04	1.60E+05	2.71E+05	2.13E+05	1.71E+05	1.13E+06	1.83E+06	9.82E+05	9.67E+04	6.88E+04	7.15E+04
pmb0592	7.71E+02	4.63E+02	3.92E+00	7.70E+02	[M+H]+	-	Chrysoeriol-O-hexosyl-O-rutinoside	Flavone	2.24E+07	2.64E+07	2.17E+07	2.20E+07	2.25E+07	2.69E+07	3.19E+07	3.79E+07	3.02E+07	9.00E+00	9.00E+00	9.00E+00	5.46E+07	4.82E+07	4.96E+07
pmb0595	4.79E+02	3.17E+02	4.17E+00	4.78E+02	[M+H]+	-	Isorhamnetin-5-O-hexoside	Flavonol	4.47E+06	6.36E+06	5.30E+06	3.91E+06	3.74E+06	3.75E+06	1.91E+07	1.00E+07	1.08E+07	2.51E+06	3.05E+06	3.54E+06	5.35E+06	3.39E+06	5.03E+06
pmb0600	6.09E+02	3.01E+02	4.08E+00	6.08E+02	[M+H]+	-	Chrysoeriol-7-O-rutinoside	Flavone	2.63E+06	3.06E+06	2.96E+06	1.57E+06	1.41E+06	2.41E+06	2.31E+06	2.69E+06	2.02E+06	6.98E+06	6.13E+06	4.70E+06	7.67E+04	7.54E+04	7.68E+04
pmb0602	5.09E+02	3.47E+02	4.17E+00	5.08E+02	[M+H]+	-	Syringetin-7-O-hexoside	Flavone	5.17E+06	6.46E+06	5.64E+06	5.39E+06	5.28E+06	7.04E+06	9.86E+06	7.67E+06	6.51E+06	4.89E+06	4.39E+06	4.53E+06	9.07E+06	8.94E+06	1.12E+07
pmb0605	4.33E+02	2.71E+02	4.21E+00	4.32E+02	[M+H]+	C04608	Apigenin-7-O-glucoside-(Cosmosiin)	Flavone	2.12E+05	1.71E+05	1.77E+05	1.35E+05	1.41E+05	2.09E+05	2.38E+05	3.16E+05	3.45E+05	1.42E+05	1.81E+05	1.43E+05	9.00E+00	9.00E+00	9.00E+00
pmb0607	4.63E+02	3.01E+02	4.30E+00	4.62E+02	[M+H]+	-	Chrysoeriol-7-O-hexoside	Flavone	1.96E+06	2.74E+06	2.77E+06	2.15E+06	1.89E+06	2.51E+06	3.74E+06	4.06E+06	4.56E+06	2.71E+06	3.87E+06	2.87E+06	1.60E+06	1.06E+06	1.47E+06
pmb0615	7.89E+02	6.27E+02	2.58E+00	7.88E+02	[M+H]+	-	Hesperetin-C-hexosyl-O-hexosyl-O-hexoside	Flavone	9.99E+04	8.96E+04	7.61E+04	8.51E+04	4.43E+04	4.84E+04	1.52E+05	7.72E+04	1.01E+05	2.61E+05	3.72E+05	3.12E+05	9.00E+00	9.00E+00	9.00E+00
pmb0620	7.87E+02	6.25E+02	2.77E+00	7.86E+02	[M+H]+	-	Chrysoeriol-6-C-hexoside-8-C-hexoside-O-hexoside	Flavone	6.31E+05	7.42E+05	7.52E+05	8.38E+05	4.84E+05	5.93E+05	9.93E+05	3.60E+05	3.18E+05	2.33E+05	3.42E+05	6.35E+05	2.78E+06	2.62E+06	2.52E+06
pmb0628	6.13E+02	4.51E+02	3.09E+00	6.12E+02	[M+H]+	-	Eriodictiol-C-hexosyl-O-hexoside	Flavone	1.69E+05	1.24E+05	1.30E+05	1.94E+05	2.14E+05	1.84E+05	9.87E+04	1.83E+05	1.04E+05	1.74E+05	1.34E+05	1.73E+05	1.96E+05	2.21E+05	2.04E+05
pmb0629	4.63E+02	3.13E+02	3.04E+00	4.62E+02	[M+H]+	-	Chrysoeriol-6-C-hexoside	Flavone	6.76E+04	4.43E+04	4.86E+04	7.60E+04	4.83E+04	7.23E+04	7.22E+04	1.07E+05	1.00E+05	4.01E+04	4.31E+04	4.04E+04	9.00E+00	9.00E+00	9.00E+00
pmb0660	7.57E+02	1.47E+02	3.73E+00	7.56E+02	[M+H]+	-	C-hexosyl-luteolin-O-p-coumaroylhexoside	Flavone	1.81E+05	2.49E+05	1.98E+05	4.52E+04	4.32E+04	5.81E+04	1.05E+05	9.10E+04	7.89E+04	9.00E+00	9.00E+00	9.00E+00	1.08E+06	1.18E+06	1.16E+06
pmb0662	7.87E+02	4.63E+02	3.64E+00	7.86E+02	[M+H]+	-	C-hexosyl-luteolin-O-feruloylhexoside	Flavone	7.63E+05	8.32E+05	7.28E+05	8.06E+05	9.34E+05	1.15E+06	1.41E+06	9.91E+05	9.80E+05	9.00E+00	9.00E+00	9.00E+00	3.78E+06	3.12E+06	3.81E+06
pmb0665	6.11E+02	4.65E+02	3.78E+00	6.10E+02	[M+H]+	-	Luteolin-8-C-hexosyl-O-hexoside	Flavone	3.52E+05	4.43E+05	3.35E+05	2.79E+05	2.90E+05	3.88E+05	3.67E+05	3.35E+05	2.31E+05	7.39E+05	8.95E+05	8.71E+05	9.00E+00	9.00E+00	9.00E+00
pmb0666	8.01E+02	6.39E+02	3.82E+00	8.00E+02	[M+H]+	-	6-C-hexosyl-apigenin-O-sinapoylhexoside	Flavone	1.97E+06	2.62E+06	2.02E+06	2.41E+06	2.83E+06	3.46E+06	3.51E+06	3.89E+06	3.57E+06	9.00E+00	9.00E+00	9.00E+00	4.78E+06	3.77E+06	4.18E+06
pmb0672	7.71E+02	4.63E+02	4.02E+00	7.70E+02	[M+H]+	-	6-C-hexosyl-apigenin-O-feruloylhexoside	Flavone	5.47E+06	7.48E+06	6.01E+06	5.03E+06	5.31E+06	7.24E+06	9.83E+06	9.66E+06	8.49E+06	9.00E+00	9.00E+00	9.00E+00	1.55E+07	1.45E+07	1.59E+07
pmb0682	8.01E+02	3.01E+02	4.31E+00	8.00E+02	[M+H]+	-	8-C-hexosyl-apigenin-O-sinapoylhexoside	Flavone	8.38E+04	8.36E+04	8.04E+04	9.00E+00	9.00E+00	4.58E+04	7.41E+04	6.90E+04	4.61E+04	9.00E+00	9.00E+00	9.00E+00	4.60E+05	2.49E+05	4.47E+05
pmb0689	4.63E+02	3.31E+02	4.75E+00	4.62E+02	[M+H]+	-	Chrysoeriol-C-hexoside	Flavone	6.53E+04	1.23E+05	8.89E+04	7.69E+04	7.94E+04	1.88E+05	2.80E+05	3.20E+05	2.87E+05	9.00E+00	9.00E+00	9.00E+00	9.00E+00	9.00E+00	9.00E+00
pmb0696	6.25E+02	4.63E+02	3.46E+00	6.24E+02	[M+H]+	-	8-C-hexosyl-chrysoeriol-O-hexoside	Flavone	9.16E+05	6.85E+05	7.50E+05	2.40E+06	1.72E+06	2.24E+06	1.46E+06	2.60E+06	1.22E+06	9.00E+00	9.00E+00	9.00E+00	7.16E+05	7.79E+05	5.96E+05
pmb0706	7.13E+02	4.65E+02	2.96E+00	7.12E+02	[M+H]+	-	Quercetin-5-O-malonylhexosyl-hexoside	Flavonol	1.93E+04	1.84E+04	1.56E+04	9.00E+00	9.00E+00	9.00E+00	9.00E+00	9.00E+00	9.00E+00	9.00E+00	9.00E+00	9.00E+00	8.98E+04	6.93E+04	9.31E+04
pmb0711	6.11E+02	3.03E+02	3.70E+00	6.10E+02	[M+H]+	-	Quercetin-7-O-rutinoside	Flavonol	5.77E+06	8.91E+06	6.79E+06	6.31E+06	5.20E+06	8.47E+06	1.07E+07	9.53E+06	6.47E+06	1.00E+07	1.41E+07	1.15E+07	1.92E+06	2.89E+06	2.47E+06
pmb0712	6.55E+02	3.31E+02	3.50E+00	6.54E+02	[M+H]+	-	Tricin-5-O-hexosyl-O-hexoside	Flavone	1.40E+06	1.48E+06	1.40E+06	1.59E+06	1.51E+06	2.41E+06	2.57E+06	2.34E+06	1.77E+06	9.00E+00	9.00E+00	9.00E+00	3.26E+06	2.59E+06	3.22E+06
pmb0713	6.55E+02	3.31E+02	3.37E+00	6.54E+02	[M+H]+	-	Tricin-7-O-hexosyl-O-hexoside	Flavone	1.34E+06	1.38E+06	1.27E+06	1.59E+06	1.50E+06	2.53E+06	2.32E+06	2.25E+06	1.56E+06	9.00E+00	9.00E+00	9.00E+00	3.22E+06	2.54E+06	3.04E+06
pmb0714	6.89E+02	3.31E+02	4.01E+00	6.88E+02	[M+H]+	-	Tricin-4'-O-(β-guaiacylglyceryl)-ether-5-O-hexoside	Flavone	9.30E+05	1.94E+06	1.84E+06	1.06E+06	8.74E+05	1.95E+06	8.83E+05	1.83E+06	8.61E+05	2.95E+06	3.10E+06	2.74E+06	2.13E+05	4.44E+04	8.28E+04
pmb0719	6.59E+02	3.31E+02	4.50E+00	6.58E+02	[M+H]+	-	Tricin-4'-O-(syringyl-alcohol)-ether-5-O-hexoside	Flavone	2.83E+05	3.74E+05	3.04E+05	8.51E+04	8.44E+04	2.08E+05	5.64E+04	1.09E+05	6.37E+04	1.65E+06	1.68E+06	1.45E+06	9.00E+00	9.00E+00	9.00E+00
pmb0725	6.69E+02	3.31E+02	5.10E+00	6.68E+02	[M+H]+	-	Tricin-7-O-feruloylhexoside	Flavone	1.38E+05	1.98E+05	1.70E+05	8.65E+04	7.87E+04	1.70E+05	1.51E+05	1.38E+05	1.24E+05	3.28E+05	2.87E+05	2.23E+05	9.00E+00	9.00E+00	1.31E+04
pmb0732	6.69E+02	3.31E+02	3.72E+00	6.68E+02	[M+H]+	-	Tricin-5-O-feruloylhexoside	Flavone	9.15E+04	2.33E+05	1.35E+05	9.00E+00	1.20E+05	1.09E+05	1.85E+05	1.69E+05	1.10E+05	1.10E+05	1.33E+05	9.00E+00	3.22E+05	1.80E+05	2.91E+05
pmb0736	4.93E+02	3.31E+02	4.30E+00	4.92E+02	[M+H]+	-	Tricin-7-O-hexoside	Flavone	1.53E+06	1.96E+06	1.47E+06	1.19E+06	1.43E+06	2.19E+06	1.68E+06	2.03E+06	3.35E+06	1.99E+06	2.00E+06	8.73E+05	1.48E+05	1.10E+05	9.00E+00
pmb0739	6.59E+02	3.31E+02	4.92E+00	6.58E+02	[M+H]+	-	Tricin-O-hexosyl-O-syringin-alcohol	Flavone	4.86E+04	9.03E+04	7.19E+04	2.07E+04	1.99E+04	3.52E+04	1.44E+04	1.97E+04	2.07E+04	3.33E+05	3.60E+05	2.74E+05	9.00E+00	9.00E+00	9.00E+00
pmb0746	5.27E+02	3.31E+02	5.84E+00	5.26E+02	[M+H]+	-	Tricin-4'-O-β-guaiacylglycerol	Flavone	8.55E+03	1.59E+04	1.23E+04	8.64E+03	9.01E+03	9.70E+03	1.96E+04	2.69E+04	2.44E+04	8.50E+03	1.32E+04	1.43E+04	9.00E+00	7.64E+03	1.20E+04
pmb0837	5.77E+02	4.25E+02	2.92E+00	5.76E+02	[M+H]+	-	Procyanidin-A3	Proanthocyanidins	1.69E+05	1.59E+05	1.48E+05	3.32E+05	3.63E+05	1.95E+05	1.84E+05	2.47E+05	2.62E+05	8.84E+04	8.77E+04	1.07E+05	9.00E+00	9.00E+00	9.00E+00
pmb2831	3.15E+02	1.53E+02	2.44E+00	3.16E+02	[M-H]-	-	Protocatechuic-acid-O-glucoside	Polyphenol	6.20E+07	6.70E+07	7.40E+07	1.07E+08	1.17E+08	1.15E+08	7.16E+07	1.30E+08	1.55E+08	4.54E+07	3.58E+07	4.67E+07	2.59E+06	2.90E+06	2.82E+06
pmb2850	3.29E+02	3.14E+02	5.73E+00	3.30E+02	[M-H]-	-	Tricin	Flavone	6.60E+04	1.15E+05	1.09E+05	3.28E+04	4.09E+04	6.60E+04	8.84E+04	1.08E+05	1.19E+05	1.20E+05	1.36E+05	1.06E+05	1.13E+05	9.09E+04	1.31E+05
pmb2947	8.65E+02	4.07E+02	3.44E+00	8.66E+02	[M-H]-	-	Catechin-catechin-catechin	Polyphenol	7.64E+05	8.47E+05	9.60E+05	1.26E+06	1.22E+06	1.44E+06	1.23E+06	2.21E+06	2.34E+06	6.38E+05	5.33E+05	6.14E+05	1.89E+05	2.44E+05	2.40E+05
pmb2954	7.71E+02	6.09E+02	2.83E+00	7.72E+02	[M-H]-	-	Luteolin-O-hexosyl-O-hexosyl-O-hexoside	Flavone	2.16E+04	2.40E+04	3.04E+04	9.00E+00	9.00E+00	9.00E+00	9.00E+00	9.00E+00	9.00E+00	4.18E+04	5.09E+04	5.03E+04	6.91E+04	6.36E+04	1.05E+05
pmb2957	4.65E+02	2.85E+02	2.59E+00	4.66E+02	[M-H]-	-	Cyanidin-O-syringic-acid	Anthocyanins	4.87E+07	6.44E+07	6.50E+07	1.01E+08	6.77E+07	7.84E+07	1.31E+08	1.14E+08	9.61E+07	9.19E+07	1.27E+08	1.01E+08	9.00E+00	9.00E+00	9.00E+00
pmb2970	6.25E+02	5.65E+02	2.19E+00	6.26E+02	[M-H]-	-	Hesperetin-O-hexosyl-O-hexoside	Flavanone	2.44E+03	2.19E+03	2.93E+03	1.93E+03	3.67E+03	2.81E+03	4.28E+03	3.45E+03	5.23E+03	5.35E+03	9.86E+03	5.84E+03	2.10E+03	2.03E+03	2.04E+03
pmb2973	7.40E+02	4.31E+02	3.34E+00	7.41E+02	[M-H]-	-	Chrysoeriol-6-C-pentosyl-O-rutinoside	Flavone	4.07E+03	3.75E+03	5.13E+03	8.82E+03	8.79E+03	7.92E+03	6.36E+03	1.30E+04	1.14E+04	9.00E+00	1.46E+03	1.09E+03	9.00E+00	9.00E+00	9.00E+00
pmb2976	7.55E+02	4.47E+02	3.74E+00	7.56E+02	[M-H]-	-	Chrysoeriol-C-pentosyl-O-hexosyl-O-hexoside	Flavone	1.26E+05	1.17E+05	1.36E+05	2.29E+04	1.79E+04	1.77E+04	1.96E+04	1.80E+04	1.17E+04	9.00E+00	9.00E+00	9.00E+00	3.14E+05	3.42E+05	3.29E+05
pmb2977	7.39E+02	4.31E+02	3.87E+00	7.40E+02	[M-H]-	-	Chrysoeriol-8-C-pentosyl-O-rutinoside	Flavone	2.02E+04	1.79E+04	1.78E+04	3.65E+04	3.01E+04	3.22E+04	2.73E+04	4.91E+04	3.71E+04	6.59E+03	9.00E+00	9.00E+00	1.80E+04	2.97E+04	9.00E+00
pmb2979	5.49E+02	3.87E+02	3.91E+00	5.50E+02	[M-H]-	-	Hesperetin-O-malonylhexoside	Flavanone	1.09E+05	1.00E+05	1.16E+05	2.70E+05	1.51E+05	2.33E+05	4.43E+05	3.06E+05	1.79E+05	9.00E+00	9.00E+00	9.00E+00	7.38E+04	7.07E+04	9.00E+00
pmb2984	4.91E+02	2.87E+02	5.09E+00	4.92E+02	[M-H]-	-	Acetyl-eriodictyol-O-hexoside	Flavone	1.31E+04	2.61E+04	3.16E+04	9.00E+00	9.00E+00	2.43E+03	6.23E+03	9.00E+00	9.00E+00	4.22E+04	6.46E+04	4.30E+04	4.47E+04	3.14E+04	5.09E+04
pmb2986	7.09E+02	5.47E+02	2.42E+00	7.10E+02	[M-H]-	-	Chrysoeriol-O-hexosyl-O-malonylhexoside	Flavone	5.28E+03	6.23E+03	7.87E+03	5.17E+03	6.54E+03	4.45E+03	6.55E+03	5.98E+03	6.25E+03	9.00E+00	9.00E+00	9.00E+00	2.90E+04	2.42E+04	3.59E+04
pmb2987	4.87E+02	1.63E+02	2.63E+00	4.88E+02	[M-H]-	-	Acacetin-O-acetyl-hexoside	Flavone	7.63E+05	8.57E+05	8.98E+05	1.18E+06	1.18E+06	1.36E+06	1.06E+06	1.45E+06	1.46E+06	1.09E+06	1.03E+06	1.11E+06	4.66E+05	4.11E+05	4.64E+05
pmb2988	6.24E+02	2.86E+02	3.15E+00	6.25E+02	[M-H]-	-	Luteolin-O-hexosyl-O-gluconic-acid	Flavone	1.33E+04	2.36E+04	2.40E+04	8.58E+03	5.45E+03	1.36E+04	1.64E+04	2.50E+04	3.17E+04	2.25E+04	4.11E+04	3.27E+04	9.00E+00	9.00E+00	9.00E+00
pmb2991	7.39E+02	2.69E+02	3.79E+00	7.40E+02	[M-H]-	-	Apigenin-O-hexosyl-O-rutinoside	Flavone	2.04E+04	2.76E+04	2.33E+04	3.66E+04	3.65E+04	4.91E+04	4.23E+04	6.36E+04	6.39E+04	9.00E+00	9.00E+00	4.88E+03	3.95E+04	3.50E+04	3.36E+04
pmb2999	4.61E+02	2.99E+02	3.87E+00	4.62E+02	[M-H]-	-	Chrysoeriol-5-O-hexoside	Flavone	1.94E+06	8.08E+05	1.17E+06	1.96E+06	1.98E+06	1.00E+06	1.48E+06	9.50E+05	1.39E+06	1.04E+06	7.87E+05	8.66E+05	8.04E+05	3.96E+05	9.27E+05

pmb3013	5.19E+02	3.14E+02	4.32E+00	5.20E+02	[M-H]-	-	Isorhamnetin-O-acetyl-hexoside	Flavonol	5.90E+04	4.58E+04	8.53E+04	5.18E+04	5.60E+04	1.86E+04	1.98E+05	1.14E+05	1.20E+05	1.33E+05	9.00E+00	1.61E+05	9.00E+00	9.00E+00	9.00E+00
pmb3023	4.49E+02	2.87E+02	3.37E+00	4.50E+02	[M-H]-	-	Eriodictyol-C-hexoside	Flavone	6.13E+06	7.00E+06	8.11E+06	3.76E+06	3.80E+06	4.32E+06	5.79E+06	6.38E+06	6.23E+06	1.20E+07	9.84E+06	1.06E+07	8.64E+06	8.03E+06	1.03E+07
pmb3024	4.47E+02	3.27E+02	3.45E+00	4.48E+02	[M-H]-	-	Luteolin-C-hexoside	Flavone	7.48E+03	8.73E+03	8.39E+03	1.35E+04	1.26E+04	1.94E+04	2.51E+04	1.01E+04	2.12E+04	9.00E+00	9.00E+00	9.00E+00	9.00E+00	9.00E+00	9.00E+00
pmb3026	5.05E+02	3.01E+02	3.80E+00	5.06E+02	[M-H]-	-	Quercetin-O-acetylhexoside	Flavonol	3.87E+05	6.21E+05	6.56E+05	2.71E+05	2.11E+05	2.44E+05	1.01E+06	7.55E+05	6.21E+05	7.00E+05	1.18E+06	1.16E+06	5.37E+04	3.90E+04	5.08E+04
pmb3041	5.21E+02	3.29E+02	3.81E+00	5.22E+02	[M-H]-	-	Tricin-O-saccharic-acid	Flavone	1.23E+06	1.43E+06	1.74E+06	9.99E+05	8.07E+05	1.02E+06	9.15E+05	1.03E+06	9.30E+05	8.89E+05	8.18E+05	9.92E+05	3.44E+06	4.15E+06	5.56E+06
pmb3042	4.91E+02	3.29E+02	4.00E+00	4.92E+02	[M-H]-	-	Tricin-5-O-hexoside	Flavone	1.11E+06	5.43E+05	5.92E+05	1.30E+06	1.07E+06	1.09E+06	9.05E+05	6.02E+05	7.87E+05	8.35E+05	7.48E+05	6.81E+05	4.91E+05	4.24E+05	5.00E+05
pmb3043	6.37E+02	3.29E+02	4.07E+00	6.38E+02	[M-H]-	-	Tricin-5-O-rutinoside	Flavone	2.39E+07	2.60E+07	2.88E+07	2.16E+07	1.92E+07	2.64E+07	1.78E+07	2.24E+07	1.93E+07	4.15E+07	3.99E+07	3.88E+07	8.59E+05	8.78E+05	8.85E+05
pmb3044	6.53E+02	3.29E+02	4.16E+00	6.54E+02	[M-H]-	-	Tricin-di-O-hexoside	Flavone	3.43E+05	3.21E+05	3.24E+05	2.59E+05	2.78E+05	2.73E+05	2.19E+05	1.18E+05	1.58E+05	1.36E+06	1.19E+06	1.24E+06	3.62E+04	9.00E+00	6.61E+04
pmb3114	5.61E+02	2.89E+02	3.61E+00	5.62E+02	[M-H]-	-	Epicatechin-epiafzelechin	Polyphenol	1.16E+04	1.35E+04	1.23E+04	1.18E+04	1.38E+04	1.70E+04	1.28E+04	1.23E+04	9.00E+00	9.00E+00	9.00E+00	9.00E+00	1.46E+04	2.05E+04	2.09E+04
pmb3894	3.29E+02	2.29E+02	5.91E+00	3.30E+02	[M-H]-	-	Di-O-methylquercetin	Flavonol	2.68E+05	4.15E+05	4.48E+05	1.53E+05	1.83E+05	2.11E+05	2.40E+05	1.92E+05	1.60E+05	2.32E+06	2.04E+05	1.63E+05	1.36E+05	1.10E+05	1.50E+05
pmc1990	2.99E+02	2.23E+02	6.78E+00	3.00E+02	[M-H]-	-	"4'-Hydroxy-5,7-dimethoxyflavanone"	Flavanone	1.68E+05	2.53E+05	3.00E+05	2.31E+04	5.62E+04	2.94E+04	1.74E+04	2.38E+04	2.04E+04	1.61E+04	1.35E+04	1.91E+04	3.52E+04	1.83E+04	2.01E+04
pme0088	2.85E+02	1.51E+02	5.00E+00	2.86E+02	[M-H]-	C01514	Luteolin	Flavone	7.78E+03	8.93E+03	1.38E+04	5.61E+03	7.47E+03	1.23E+04	2.02E+04	8.13E+03	1.35E+04	7.29E+03	9.00E+00	9.00E+00	9.00E+00	9.00E+00	9.00E+00
pme0094	4.47E+02	2.85E+02	2.61E+00	4.48E+02	[M-H]-	C08604	Cyanidin-3-O-glucoside-(Kuromanin)	Anthocyanins	7.00E+06	7.95E+06	8.09E+06	1.25E+07	8.37E+06	9.72E+06	1.36E+07	1.30E+07	1.19E+07	1.31E+07	1.65E+07	1.39E+07	9.00E+00	9.00E+00	9.00E+00
pme0197	6.09E+02	3.01E+02	3.70E+00	6.10E+02	[M-H]-	C05625	Quercetin-3-O-rutinoside-(Rutin)	Flavonol	1.59E+07	1.94E+07	1.99E+07	7.50E+06	6.35E+06	9.12E+06	1.01E+07	1.04E+07	8.55E+06	4.07E+07	5.51E+07	5.23E+07	1.32E+06	2.38E+06	2.08E+06
pme0199	3.01E+02	1.51E+02	5.12E+00	3.02E+02	[M-H]-	C00389	Quercetin	Flavonol	1.29E+06	2.44E+06	2.54E+06	1.15E+06	7.68E+05	1.79E+06	2.60E+06	2.87E+06	3.29E+06	1.30E+06	5.87E+05	6.42E+05	3.00E+05	3.47E+05	4.50E+05
pme0200	2.87E+02	1.53E+02	5.73E+00	2.86E+02	[M+H]+	C05903	Kaempferol	Flavonol	2.28E+04	2.13E+04	3.30E+04	1.58E+04	8.89E+03	1.58E+04	5.39E+04	3.03E+04	5.79E+04	9.64E+03	7.92E+03	1.25E+04	9.00E+00	9.00E+00	9.00E+00
pme0201	2.91E+02	1.39E+02	2.99E+00	2.90E+02	[M+H]+	C06562	Catechin	Polyphenol	1.69E+07	1.79E+07	1.63E+07	1.73E+07	1.61E+07	1.71E+07	1.81E+07	3.07E+07	2.88E+07	1.89E+07	1.86E+07	1.97E+07	1.64E+06	1.48E+06	2.89E+06
pme0331	5.81E+02	2.73E+02	4.16E+00	5.80E+02	[M+H]+	C09789	Naringenin-7-O-neohesperidoside-(Naringin)	Flavanone	2.19E+05	2.30E+05	2.27E+05	4.78E+04	6.44E+04	5.55E+04	5.72E+04	6.04E+04	6.35E+04	6.30E+05	6.71E+05	4.18E+05	9.00E+00	9.00E+00	9.00E+00
pme0333	5.79E+02	2.71E+02	4.01E+00	5.78E+02	[M+H]+	C12627	Apigenin-7-O-neohesperidoside-(Rhoifolin)	Flavone	9.42E+06	1.02E+07	9.29E+06	2.35E+06	2.24E+06	2.82E+06	3.45E+06	3.17E+06	1.92E+06	2.63E+07	2.71E+07	2.30E+07	6.63E+05	8.23E+05	9.66E+05
pme0361	4.35E+02	3.03E+02	3.98E+00	4.34E+02	[M+H]+	-	Quercetin-3-alpha-L-arabinofuranoside-(Avicularin)	Flavonol	7.74E+06	1.16E+07	8.23E+06	4.79E+05	3.77E+05	3.72E+05	2.23E+06	1.01E+06	1.37E+06	1.78E+05	2.67E+05	3.49E+05	1.15E+08	7.54E+07	9.44E+07
pme0363	2.99E+02	2.84E+02	5.76E+00	3.00E+02	[M-H]-	C04293	Chrysoeriol	Flavone	5.33E+04	7.98E+04	7.60E+04	2.03E+04	1.52E+04	4.42E+04	1.55E+05	9.60E+04	9.52E+04	6.28E+04	3.92E+04	4.32E+04	2.96E+04	3.31E+04	6.67E+04
pme0368	5.79E+02	2.71E+02	4.01E+00	5.78E+02	[M+H]+	-	Apigenin-7-rutinoside-(Isorhoifolin)	Flavone	8.86E+06	1.08E+07	8.98E+06	2.48E+06	2.26E+06	2.74E+06	3.82E+06	3.44E+06	2.20E+06	2.68E+07	2.78E+07	2.28E+07	6.72E+05	8.43E+05	8.81E+05
pme0369	5.93E+02	2.85E+02	3.83E+00	5.94E+02	[M-H]-	-	Kaempferol-3-O-rutinoside-(Nicotiflorin)	Flavonol	4.94E+06	5.88E+06	5.64E+06	4.30E+06	3.38E+06	4.65E+06	5.27E+06	4.58E+06	3.33E+06	1.08E+07	1.31E+07	1.15E+07	9.56E+05	1.42E+06	1.36E+06
pme0371	4.33E+02	2.71E+02	4.22E+00	4.34E+02	[M-H]-	C09099	Naringenin-7-O-glucoside-(Prunin)	Flavanone	1.19E+06	1.36E+06	1.67E+06	9.88E+05	9.06E+05	1.05E+06	1.90E+06	1.79E+06	2.15E+06	1.02E+06	1.30E+06	1.25E+06	1.38E+06	1.63E+06	1.86E+06
pme0377	2.73E+02	1.53E+02	5.59E+00	2.72E+02	[M+H]+	C00509	Naringenin	Flavanone	2.87E+06	3.88E+06	3.56E+06	1.65E+06	1.55E+06	2.77E+06	6.30E+06	8.31E+06	9.75E+06	2.90E+06	2.60E+06	3.56E+06	1.55E+06	1.80E+06	2.62E+06
pme0379	2.71E+02	1.53E+02	5.63E+00	2.70E+02	[M+H]+	C01477	Apigenin	Flavone	1.91E+04	2.50E+04	2.53E+04	1.71E+04	7.09E+03	1.22E+04	5.55E+04	3.24E+04	5.54E+04	2.31E+04	2.18E+04	2.30E+04	9.00E+00	9.00E+00	1.03E+04
pme0421	2.75E+02	1.69E+02	5.56E+00	2.74E+02	[M+H]+	C00774	Phloretin	Flavanone	3.73E+04	3.90E+04	5.21E+04	7.52E+03	6.36E+03	1.87E+04	1.62E+04	2.66E+04	3.25E+04	1.92E+04	4.12E+04	4.74E+04	3.54E+04	2.03E+05	4.78E+04
pme0434	5.77E+02	4.07E+02	3.03E+00	5.78E+02	[M-H]-	-	Procyanidin-B2	Proanthocyanidins	4.68E+06	5.05E+06	5.41E+06	5.21E+06	4.47E+06	4.66E+06	5.32E+06	7.46E+06	7.38E+06	1.39E+06	1.15E+06	1.08E+06	8.37E+06	1.04E+07	8.69E+06
pme0436	5.77E+02	4.07E+02	2.79E+00	5.78E+02	[M-H]-	-	Procyanidin-B3	Proanthocyanidins	2.48E+07	2.50E+07	2.69E+07	3.59E+07	3.84E+07	3.78E+07	3.03E+07	4.50E+07	4.96E+07	2.28E+07	1.92E+07	2.15E+07	1.94E+05	2.18E+05	2.06E+05
pme0460	2.91E+02	1.39E+02	3.32E+00	2.90E+02	[M+H]+	C09727	L-Epicatechin	Polyphenol	2.27E+07	2.33E+07	2.25E+07	3.45E+07	2.86E+07	3.38E+07	3.29E+07	5.92E+07	4.54E+07	2.53E+07	2.76E+07	2.86E+07	7.57E+06	1.26E+07	8.77E+06
pme1399	3.55E+02	1.79E+02	8.40E+00	3.54E+02	[M+H]+	C16417	Xanthohumol	Flavanone	6.97E+03	1.02E+04	8.01E+03	1.78E+03	3.68E+03	4.35E+03	5.29E+03	3.78E+03	3.43E+03	3.46E+04	8.18E+03	7.42E+03	1.27E+04	8.66E+03	1.57E+04
pme1480	3.19E+02	1.53E+02	4.70E+00	3.18E+02	[M+H]+	C10107	Myricetin	Flavonol	1.24E+06	2.06E+06	1.50E+06	1.55E+06	8.38E+05	1.92E+06	2.81E+06	1.53E+06	1.13E+06	3.28E+05	5.48E+05	6.22E+05	5.41E+05	2.28E+06	1.51E+06
pme1486	4.57E+02	1.69E+02	3.33E+00	4.58E+02	[M-H]-	C09731	Epigallate-catechin-gallate-(EGCG)	Polyphenol	1.28E+06	1.54E+06	1.82E+06	1.47E+06	1.08E+06	1.64E+06	1.87E+06	2.46E+06	2.36E+06	9.75E+05	9.37E+05	1.66E+06	1.26E+06	1.04E+06	1.33E+06
pme1500	3.13E+02	2.83E+02	7.23E+00	3.14E+02	[M-H]-	-	Kumatakenin	Flavonol	6.93E+02	1.62E+03	1.30E+03	1.06E+03	5.39E+02	1.49E+03	2.81E+03	3.30E+03	2.38E+03	9.00E+00	9.00E+00	9.00E+00	9.00E+00	6.24E+02	9.00E+00
pme1506	4.33E+02	2.87E+02	4.94E+00	4.32E+02	[M+H]+	-	Kaempferol-7-O-rhamnoside	Flavonol	7.24E+04	1.29E+05	9.50E+04	4.81E+04	6.85E+04	8.02E+04	8.82E+04	1.03E+05	1.17E+05	7.12E+04	1.22E+05	1.03E+05	9.00E+00	6.07E+04	5.86E+04
pme1516	3.07E+02	1.39E+02	2.76E+00	3.06E+02	[M+H]+	C12136	Epigallocatechin-(EGC)	Polyphenol	3.76E+06	3.99E+06	3.78E+06	2.06E+06	1.23E+06	1.55E+06	1.72E+06	2.23E+06	1.56E+06	3.03E+06	3.82E+06	4.11E+06	5.24E+06	1.97E+07	6.19E+06
pme1518	4.03E+02	3.73E+02	7.06E+00	4.02E+02	[M+H]+	C10112	Nobiletin	Flavone	5.13E+05	7.80E+05	7.20E+05	4.76E+05	5.89E+05	1.01E+06	2.65E+05	3.07E+05	1.77E+06	1.85E+05	8.62E+05	1.24E+06	2.04E+05	4.43E+05	6.24E+05
pme1524	3.05E+02	2.31E+02	4.14E+00	3.04E+02	[M+H]+	C01617	Dihydroquercetin-(Taxifolin)	Flavonol	9.16E+04	1.26E+05	1.06E+05	9.00E+00	9.00E+00	9.00E+00	9.00E+00	9.00E+00	9.00E+00	2.78E+05	3.58E+05	3.82E+05	9.00E+00	4.81E+04	9.00E+00
pme1537	3.07E+02	1.39E+02	2.27E+00	3.06E+02	[M+H]+	C12127	(+)-Gallocatechin-(GC)	Polyphenol	6.53E+06	6.98E+06	6.50E+06	1.03E+07	8.42E+06	1.07E+07	8.63E+06	1.84E+07	1.57E+07	1.45E+07	1.55E+07	1.60E+07	4.37E+04	2.07E+05	8.17E+04
pme1540	6.25E+02	3.17E+02	3.66E+00	6.24E+02	[M+H]+	-	Isorhamnetin-3-O-neohesperidoside	Flavonol	4.44E+06	7.05E+06	4.92E+06	4.94E+06	4.49E+06	7.83E+06	6.32E+06	7.31E+06	5.77E+06	7.75E+06	8.37E+06	7.71E+06	9.73E+05	8.60E+05	6.63E+05
pme1544	2.85E+02	2.70E+02	7.06E+00	2.84E+02	[M+H]+	C01470	Acacetin	Flavone	4.96E+03	9.73E+03	9.11E+03	9.00E+00	9.00E+00	9.00E+00	9.00E+00	9.00E+00	9.00E+00	9.00E+00	2.70E+04	1.67E+04	3.97E+04	2.84E+03	1.64E+04
pme1550	3.73E+02	3.43E+02	7.54E+00	3.72E+02	[M+H]+	C10190	Tangeretin	Flavone	7.34E+05	1.12E+06	9.09E+05	5.85E+05	6.81E+05	1.23E+06	3.18E+05	3.48E+05	1.92E+06	2.20E+05	1.14E+06	1.70E+06	2.35E+05	3.63E+05	7.20E+05
pme1552	4.65E+02	3.19E+02	3.63E+00	4.64E+02	[M+H]+	C10108	Myricetin-3-O-rhamnoside-(Myricitrin)	Flavonol	5.19E+05	5.83E+05	5.14E+05	9.00E+00	9.00E+00	9.00E+00	9.00E+00	9.00E+00	9.00E+00	9.00E+00	9.00E+00	1.79E+04	3.10E+06	3.63E+06	3.39E+06
pme1562	4.41E+02	1.69E+02	3.89E+00	4.42E+02	[M-H]-	-	Epicatechin-gallate-(ECG)	Polyphenol	2.71E+06	3.42E+06	4.01E+06	3.51E+06	2.97E+06	3.71E+06	2.41E+06	4.12E+06	3.91E+06	3.20E+06	3.01E+06	3.34E+06	1.45E+06	8.45E+05	2.51E+06
pme1583	2.89E+02	1.53E+02	5.05E+00	2.88E+02	[M+H]+	C05631	Eriodictyol	Flavanone	1.07E+05	1.19E+05	1.30E+05	7.27E+04	7.47E+04	1.20E+05	1.21E+05	1.93E+05	2.52E+05	1.63E+05	8.64E+04	1.08E+05	6.46E+04	7.71E+04	1.20E+05
pme1588	3.15E+02	1.51E+02	5.85E+00	3.16E+02	[M-H]-	C10084	Isorhamnetin	Flavonol	6.16E+04	1.02E+05	9.26E+04	3.37E+04	2.55E+04	5.08E+04	6.30E+04	4.57E+04	4.37E+04	8.83E+04	5.60E+04	4.32E+04	9.00E+00	9.00E+00	9.00E+00
pme1598	4.63E+02	3.01E+02	3.85E+00	4.64E+02	[M-H]-	-	Hesperetin-5-O-glucoside	Flavanone	2.66E+07	3.15E+07	3.86E+07	2.65E+07	2.51E+07	1.95E+07	5.06E+07	3.78E+07	3.97E+07	2.19E+07	2.69E+07	3.01E+07	3.05E+07	2.78E+07	3.68E+07
pme1600	3.03E+02	1.67E+02	6.28E+00	3.02E+02	[M+H]+	-	7-O-Methyleriodictyol	Flavanone	5.79E+03	7.45E+03	5.94E+03	9.00E+00	9.00E+00	9.00E+00	8.00E+03	6.02E+03	1.02E+04	1.52E+04	1.81E+04	2.02E+04	9.00E+00	5.34E+03	2.98E+03
pme1605	5.93E+02	2.85E+02	3.76E+00	5.94E+02	[M-H]-	-	Kaempferol-3-O-robinobioside-(Biorobin)	Flavonol	4.74E+06	5.18E+06	5.97E+06	4.27E+06	3.62E+06	3.87E+06	4.90E+06	3.99E+06	3.21E+06	1.06E+07	1.08E+07	1.12E+07	8.10E+05	1.37E+06	1.19E+06
pme1611	4.33E+02	3.13E+02	3.75E+00	4.34E+02	[M-H]-	-	Isohemiphloin	Flavone	5.70E+04	7.13E+04	1.06E+05	1.11E+05	1.33E+05	1.13E+05	2.05E+05	1.95E+05	2.84E+05	1.83E+04	9.35E+03	1.31E+04	9.69E+03	1.09E+04	9.58E+03
pme1622	4.47E+02	2.85E+02	3.93E+00	4.48E+02	[M-H]-	C12249	Kaempferol-3-O-glucoside-(Astragalin)	Flavonol	1.41E+06	1.51E+06	1.93E+06	1.78E+06	1.73E+06	1.99E+06	1.41E+06	2.15E+06	1.75E+06	1.98E+06	1.53E+06	1.78E+06	3.74E+05	4.79E+05	4.63E+05
pme1624	4.33E+02	2.83E+02	3.78E+00	4.32E+02	[M+H]+	C01714	Isovitexin	Flavone	2.91E+04	3.20E+04	3.24E+04	1.52E+04	4.19E+04	3.72E+04	5.59E+04	5.64E+04	7.64E+04	1.36E+04	7.99E+03	4.66E+04	9.00E+00	1.20E+04	1.29E+04
pme1667	5.95E+02	4.15E+02	3.36E+00	5.94E+02	[M+H]+	C08064	Isovitexin-7-O-glucoside-(Saponarin)	Flavone	8.09E+04	6.50E+04	6.42E+04	6.24E+04	9.00E+00	7.65E+04	9.00E+00	9.00E+00	5.44E+04	8.97E+04	9.00E+00	5.99E+04	9.00E+00	9.00E+00	9.00E+00
pme1773	5.95E+02	2.87E+02	2.70E+00	5.95E+02	Protonated	C08620	Cyanidin-3-O-rutinoside-(Keracyanin)	Anthocyanins	7.89E+04	9.41E+04	9.06E+04	1.68E+05	7.87E+04	5.74E+04	5.84E+04	1.42E+05	1.19E+05	8.04E+05	9.00E+00	9.33E+05	2.50E+02	9.00E+00	9.00E+00
pme1777	6.11E+02	2.87E+02	2.08E+00	6.11E+02	Protonated	C08639	"Cyanidin-3,5-O-diglucoside-(Cyanin)"	Anthocyanins	4.76E+06	5.21E+06	5.30E+06	1.99E+06	1.85E+06	2.15E+06	4.06E+06	3.16E+06	2.47E+06	9.00E+00	9.00E+00	9.00E+00	7.64E+06	7.19E+06	9.01E+06
pme1793	5.95E+02	2.71E+02	2.38E+00	5.95E+02	Protonated	C08725	Pelargonin	Anthocyanins	2.26E+07	2.41E+07	2.30E+07	1.55E+07	1.64E+07	1.99E+07	2.34E+07	2.04E+07	1.56E+07	9.00E+00	9.00E+00	9.00E+00	4.37E+07	4.05E+07	4.46E+07
pme1824	1.53E+02	1.09E+02	2.48E+00	1.54E+02	[M-H]-	C00230	Protocatechuic-acid	Polyphenol	1.17E+06	1.28E+06	1.47E+06	5.60E+05	5.23E+05	4.90E+05	5.62E+05	4.80E+05	4.77E+05	2.71E+06	9.16E+05	7.98E+05	1.70E+06	1.38E+06	1.57E+06
pme2246	3.01E+02	1.85E+02	3.89E+00	3.02E+02	[M-H]-	C10788	Ellagic-acid	Polyphenol	4.76E+05	9.38E+05	1.06E+06	5.39E+05	5.30E+05	8.36E+05	1.11E+06	1.98E+06	1.64E+06	8.82E+04	9.01E+04	8.13E+04	8.00E+04	4.65E+04	7.53E+04
pme2319	3.01E+02	1.64E+02	5.75E+00	3.02E+02	[M-H]-	C01709	Hesperetin	Flavanone	5.84E+03	7.93E+03	1.12E+04	3.57E+03	2.27E+03	4.01E+03	1.38E+04	1.28E+04	1.28E+04	8.79E+03	5.46E+03	7.22E+03	1.06E+04	9.97E+03	2.07E+04
pme2459	4.49E+02	2.87E+02	3.87E+00	4.48E+02	[M+H]+	C03951	Luteolin-7-O-glucoside-(Cynaroside)	Flavone	2.23E+05	2.86E+05	2.10E+05	9.00E+00	1.19E+05	1.63E+05	7.34E+05	5.32E+05	5.37E+05	9.00E+00	9.00E+00	7.31E+04	3.96E+05	3.26E+05	3.30E+05
pme2482	1.39E+02	9.28E+01	3.03E+00	1.38E+02	[M+H]+	C16700	Protocatechuic-aldehyde	Polyphenol	2.59E+05	2.74E+05	1.92E+05	2.23E+05	1.66E+05	2.66E+05	1.97E+05	3.23E+05	3.80E+05	3.62E+05	3.22E+05	3.54E+05	1.09E+05	9.69E+04	7.30E+04
pme2491	5.78E+02	2.85E+02	3.74E+00	5.78E+02	[M-H]-	C16981	"Kaempferol-3,7-dirhamnoside-(Kaempferitrin)"	Flavonol	5.57E+03	6.91E+03	4.17E+03	1.17E+04	8.67E+03	1.28E+04	8.44E+03	2.29E+04	1.70E+04	9.00E+00	2.90E+03	3.83E+03	9.00E+00	9.00E+00	9.00E+00
pme2895	3.19E+02	3.01E+02	3.52E+00	3.20E+02	[M-H]-	C02906	Dihydromyricetin	Flavonol	7.51E+04	8.00E+04	8.88E+04	4.40E+04	4.66E+04	5.93E+04	1.12E+05	1.50E+05	1.86E+05	6.67E+04	8.75E+04	1.02E+05	9.62E+04	1.22E+05	1.11E+05
pme2949	6.09E+02	3.01E+02	4.08E+00	6.10E+02	[M-H]-	C09755	Hesperetin-7-rutinoside-(Hesperidin)	Flavanone	2.17E+06	2.84E+06	2.49E+06	1.26E+06	1.76E+06	1.41E+06	1.53E+06	2.24E+06	1.38E+06	2.87E+06	5.91E+06	5.78E+06	2.52E+05	6.05E+05	4.87E+05
pme2960	2.73E+02	1.53E+02	5.57E+00	2.72E+02	[M+H]+	C06561	Naringenin-chalcone	Flavanone	2.91E+06	3.95E+06	3.70E+06	1.60E+06	1.59E+06	2.77E+06	6.39E+06	8.33E+06	9.83E+06	2.91E+06	2.68E+06	3.47E+06	1.60E+06	1.76E+06	2.71E+06
pme2963	2.87E+02	2.59E+02	4.62E+00	2.88E+02	[M-H]-	C00974	Aromadedrin-(Dihydrokaempferol)	Flavonol	5.52E+05	7.92E+05	8.74E+05	2.00E+05	2.10E+05	3.14E+05	8.27E+05	8.08E+05	9.10E+05	9.12E+05	4.55E+05	6.54E+05	7.88E+05	1.26E+06	1.97E+06
pme2973	6.75E+02	5.13E+02	4.98E+00	6.76E+02	[M-H]-	C17555	"Icariin-(kaempferol-3,7-O-diglucoside-8-prenyl-derivative)"	Flavonol	3.05E+04	3.35E+04	4.35E+04	9.00E+00	7.07E+03	1.60E+04	4.73E+04	6.36E+04	7.11E+04	7.10E+04	4.34E+04	3.19E+04	4.19E+04	1.54E+04	2.59E+04
pme2982	2.57E+02	1.53E+02	7.05E+00	2.56E+02	[M+H]+	C09827	Pinocembrin-(Dihydrochrysin)	Flavanone	3.09E+04	5.36E+04	5.01E+04	5.82E+03	8.84E+03	1.04E+04	3.11E+04	1.00E+04	8.66E+03	1.90E+04	9.00E+00	7.46E+03	1.36E+05	7.36E+04	3.76E+05
pme3129	4.63E+02	3.01E+02	3.86E+00	4.64E+02	[M-H]-	-	Quercetin-4'-O-glucoside-(Spiraeoside)	Flavonol	1.98E+07	2.33E+07	2.98E+07	2.07E+07	1.95E+07	1.46E+07	4.02E+07	3.04E+07	3.14E+07	1.66E+07	2.14E+07	2.37E+07	2.52E+07	2.16E+07	2.75E+07
pme3137	2.39E+02	1.65E+02	7.73E+00	2.38E+02	[M+H]+	C20871	3-Hydroxyflavone	Flavonol	3.35E+03	4.92E+03	5.17E+03	2.67E+03	9.00E+00	9.00E+00	5.08E+03	6.20E+03	7.43E+03	9.00E+00	9.00E+00	9.00E+00	7.87E+03	6.05E+03	6.74E+03
pme3208	4.45E+02	2.83E+02	3.54E+00	4.46E+02	[M-H]-	C16195	Glycitin	Isoflavone	3.00E+04	3.34E+04	4.58E+04	9.00E+00	9.00E+00	9.00E+00	9.00E+00	9.00E+00	9.00E+00	8.05E+04	6.89E+04	1.01E+05	7.24E+04	8.08E+04	1.24E+05
pme3210	4.33E+02	2.71E+02	4.01E+00	4.32E+02	[M+H]+	C09126	Genistein-7-O-Glucoside-(Genistin)	Isoflavone	1.54E+05	1.38E+05	8.19E+04	1.24E+05	1.20E+05	1.06E+05	2.99E+05	2.23E+05	2.44E+05	9.00E+00	9.00E+00	6.48E+04	3.46E+05	3.02E+05	3.24E+05
pme3212	4.65E+02	3.03E+02	3.87E+00	4.64E+02	[M+H]+	C05623	Quercetin-3-O-glucoside-(Isotrifoliin)	Flavonol	3.76E+05	2.84E+05	3.05E+05	2.08E+05	2.06E+05	9.00E+00	7.08E+05	6.54E+05	2.39E+05	9.00E+00	9.00E+00	9.00E+00	6.27E+05	4.19E+05	6.32E+05

pme3224	5.77E+02	4.13E+02	3.67E+00	5.78E+02	[M-H]-	C12628	Vitexin-2"-O-beta-L-rhamnoside	Flavone	9.96E+03	1.32E+04	1.43E+04	1.47E+04	1.22E+04	1.41E+04	2.24E+04	1.71E+04	1.85E+04	9.00E+00	9.00E+00	9.00E+00	3.20E+03	5.01E+03	9.00E+00
pme3233	2.85E+02	2.70E+02	5.00E+00	2.84E+02	[M+H]+	C01562	Calycosin	Isoflavone	2.97E+04	3.31E+04	3.03E+04	1.44E+04	1.68E+04	2.30E+04	4.66E+04	2.96E+04	3.77E+04	9.00E+00	9.00E+00	9.00E+00	8.25E+04	6.04E+04	8.98E+04
pme3250	2.85E+02	2.70E+02	7.00E+00	2.84E+02	[M+H]+	C00814	Biochanin-A	Isoflavone	1.00E+04	1.29E+04	1.05E+04	9.00E+00	9.00E+00	9.00E+00	9.00E+00	9.00E+00	9.00E+00	9.00E+00	3.12E+04	1.62E+04	4.15E+04	9.00E+00	1.58E+04
pme3251	2.83E+02	2.68E+02	5.13E+00	2.84E+02	[M-H]-	C14536	Glycitein	Isoflavone	2.88E+03	5.25E+03	4.00E+03	4.29E+03	2.81E+03	4.29E+03	5.41E+03	8.07E+03	7.72E+03	9.00E+00	9.00E+00	9.00E+00	9.00E+00	9.00E+00	9.00E+00
pme3263	2.69E+02	2.25E+02	4.43E+00	2.70E+02	[M-H]-	C02495	2'-Hydroxydaidzein	Isoflavone	1.76E+04	2.75E+04	3.19E+04	5.05E+03	9.90E+03	1.16E+04	2.85E+04	1.18E+04	1.02E+04	7.35E+03	1.07E+04	1.15E+04	6.07E+04	5.27E+04	5.03E+04
pme3267	4.47E+02	2.85E+02	3.86E+00	4.48E+02	[M-H]-	C12626	Kaempferol-3-O-galactoside-(Trifolin)	Flavonol	9.10E+05	1.15E+06	1.27E+06	1.03E+06	1.21E+06	1.40E+06	9.37E+05	1.48E+06	1.48E+06	1.41E+06	1.04E+06	1.34E+06	2.44E+05	3.39E+05	3.41E+05
pme3297	4.33E+02	2.87E+02	4.49E+00	4.32E+02	[M+H]+	C16911	Kaempferol-3-O-rhamnoside-(Kaempferin)	Flavonol	1.45E+05	2.18E+05	1.53E+05	9.00E+00	9.00E+00	9.00E+00	9.00E+00	9.00E+00	9.00E+00	9.00E+00	9.00E+00	9.00E+00	1.36E+06	6.37E+05	1.05E+06
pme3300	3.01E+02	1.51E+02	4.51E+00	3.02E+02	[M-H]-	C10192	Tricetin	Flavone	7.03E+04	1.65E+05	1.82E+05	5.03E+04	4.22E+04	1.30E+05	1.35E+05	1.18E+05	1.09E+05	3.54E+04	6.89E+04	6.99E+04	1.30E+05	1.53E+05	1.77E+05
pme3391	4.79E+02	3.17E+02	2.56E+00	4.79E+02	Protonated	C12139	Petunidin-3-O-glucoside	Anthocyanins	6.97E+06	8.25E+06	6.11E+06	8.53E+06	7.82E+06	1.06E+07	9.36E+06	8.30E+06	7.14E+06	1.67E+07	1.80E+07	1.47E+07	9.00E+00	9.00E+00	9.00E+00
pme3392	4.33E+02	2.71E+02	2.83E+00	4.33E+02	Protonated	-	Pelargonidin-3-O-beta-D-glucoside（Callistephin-chloride)	Anthocyanins	2.39E+07	2.65E+07	2.01E+07	1.35E+07	1.14E+07	1.29E+07	1.96E+07	1.58E+07	1.06E+07	7.57E+07	8.26E+07	6.43E+07	8.53E+05	9.63E+05	1.12E+06
pme3404	3.47E+02	1.53E+02	5.80E+00	3.46E+02	[M+H]+	C11620	Syringetin	Flavonol	1.18E+05	2.09E+05	1.49E+05	5.40E+04	3.67E+04	1.09E+05	8.37E+04	8.13E+04	6.12E+04	1.28E+05	1.59E+05	1.37E+05	2.22E+04	5.57E+04	3.98E+04
pme3407	3.31E+02	1.51E+02	5.15E+00	3.32E+02	[M-H]-	C12633	Laricitrin	Flavonol	1.21E+05	2.13E+05	2.72E+05	1.07E+05	5.47E+04	1.55E+05	1.66E+05	1.45E+05	1.32E+05	1.38E+05	9.62E+04	8.01E+04	5.21E+04	9.51E+04	1.08E+05
pme3442	4.77E+02	3.01E+02	3.76E+00	4.78E+02	[M-H]-	-	Quercetin-7-O-β-D-Glucuronide	Flavonol	1.68E+06	2.48E+06	2.92E+06	1.72E+06	1.86E+06	2.24E+06	3.78E+06	4.23E+06	4.62E+06	6.95E+05	1.07E+06	1.03E+06	9.51E+05	7.25E+05	8.62E+05
pme3461	3.01E+02	1.51E+02	5.75E+00	3.02E+02	[M-H]-	C09756	Homoeriodictyol	Flavanone	5.02E+03	7.00E+03	7.77E+03	2.66E+03	9.00E+00	9.00E+00	6.37E+03	8.83E+03	1.04E+04	3.73E+03	4.98E+03	3.29E+03	9.72E+03	1.36E+04	1.62E+04
pme3468	7.39E+02	5.93E+02	3.35E+00	7.40E+02	[M-H]-	C10178	Kaempferol-3-O-robinoside-7-O-rhamnoside-(Robinin)	Flavonol	6.02E+03	4.27E+03	6.24E+03	1.11E+04	8.43E+03	9.98E+03	1.29E+04	1.42E+04	1.46E+04	9.00E+00	9.00E+00	1.27E+03	9.00E+00	9.00E+00	9.00E+00
pme3475	2.73E+02	1.53E+02	5.59E+00	2.72E+02	[M+H]+	C09614	Butin	Flavone	2.85E+06	3.76E+06	3.67E+06	1.49E+06	1.47E+06	2.68E+06	6.07E+06	8.30E+06	9.71E+06	2.76E+06	2.59E+06	3.48E+06	1.53E+06	1.81E+06	2.64E+06
pme3484	4.79E+02	3.16E+02	3.53E+00	4.80E+02	[M-H]-	-	Myricetin-3-O-galactoside	Flavonol	1.15E+07	1.49E+07	1.64E+07	1.25E+07	1.09E+07	1.30E+07	1.74E+07	1.32E+07	1.45E+07	6.45E+06	7.43E+06	8.57E+06	2.10E+07	2.27E+07	2.21E+07
pme3502	4.29E+02	2.67E+02	4.59E+00	4.30E+02	[M-H]-	C10509	Formononetin-7-O-glucoside-(Ononin)	Isoflavone	1.62E+04	2.61E+04	3.18E+04	4.49E+03	9.00E+00	9.00E+00	9.00E+00	9.00E+00	9.00E+00	9.38E+04	9.74E+04	1.05E+05	1.99E+04	1.19E+04	1.82E+04
pmf0005	5.79E+02	2.71E+02	4.05E+00	5.81E+02	[M-H]-	C09793	Narirutin	Flavone	6.00E+04	7.31E+04	8.28E+04	6.66E+04	8.16E+04	7.40E+04	1.83E+05	1.06E+05	1.23E+05	4.01E+04	4.30E+04	5.51E+04	2.88E+04	5.05E+04	7.03E+04
pmf0011	5.93E+02	4.73E+02	3.19E+00	5.94E+02	[M-H]-	-	"Apigenin-6,8-C-diglucoside"	Flavone	2.87E+03	4.17E+03	2.43E+03	2.56E+03	3.42E+03	3.98E+03	6.22E+03	6.89E+03	7.51E+03	9.00E+00	9.00E+00	2.10E+03	9.00E+00	9.00E+00	9.00E+00
pmf0012	5.95E+02	4.57E+02	3.18E+00	5.94E+02	[M+H]+	-	"6,8-di-C-glucoside-Apigenine"	Flavone	4.79E+04	3.84E+04	4.39E+04	4.49E+04	5.38E+04	4.48E+04	4.36E+04	4.70E+04	3.95E+04	5.46E+04	4.94E+04	5.53E+04	9.00E+00	9.00E+00	9.00E+00
pmf0027	4.49E+02	2.87E+02	2.68E+00	4.48E+02	[M]+	C08647	Cyanidin-3-O-galactoside	Anthocyanins	1.96E+07	2.41E+07	2.16E+07	2.42E+07	1.91E+07	2.43E+07	4.39E+07	3.98E+07	2.50E+07	3.52E+07	4.79E+07	3.36E+07	5.92E+05	8.46E+05	9.53E+05
pmf0057	2.71E+02	1.51E+02	5.66E+00	2.72E+02	[M-H]-	C06561	"4,2',4',6'-Tetrahydroxychalcone--"	Flavone	1.22E+06	1.87E+06	1.95E+06	6.59E+05	6.22E+05	1.12E+06	2.96E+06	4.03E+06	4.60E+06	1.25E+06	1.30E+06	1.61E+06	8.06E+05	9.73E+05	1.55E+06
pmf0058	2.71E+02	1.51E+02	5.66E+00	2.72E+02	[M-H]-	C00509	"4',5,7-Trihydroxyflavanone"	Flavanone	1.11E+06	1.70E+06	1.86E+06	5.93E+05	5.60E+05	9.90E+05	2.79E+06	3.66E+06	4.14E+06	1.12E+06	1.20E+06	1.49E+06	7.36E+05	9.09E+05	1.46E+06
pmf0108	2.71E+02	2.43E+02	4.35E+00	2.72E+02	[M-H]-	C09751	Garbanzol	Flavanone	2.97E+04	2.25E+04	2.96E+04	9.00E+00	9.00E+00	9.00E+00	9.00E+00	9.00E+00	9.00E+00	9.00E+00	9.00E+00	9.00E+00	2.06E+05	2.45E+05	2.66E+05
pmf0127	4.05E+02	2.43E+02	5.09E+00	4.04E+02	[M+H]+	-	Deoxyrhapontin	Flavone	3.91E+05	6.00E+05	5.15E+05	4.31E+04	6.14E+04	8.32E+04	3.52E+05	1.36E+05	1.49E+05	6.24E+04	7.31E+04	5.40E+04	3.07E+06	1.53E+06	1.88E+06
pmf0179	6.23E+02	3.15E+02	4.04E+00	6.24E+02	[M-H]-	-	Narcissoside	Flavone	6.19E+06	6.67E+06	7.48E+06	5.20E+06	5.70E+06	7.18E+06	4.95E+06	5.01E+06	4.85E+06	1.41E+07	1.47E+07	1.48E+07	5.28E+05	7.82E+05	4.45E+05
pmf0204	4.65E+02	3.03E+02	3.73E+00	4.64E+02	[M+H]+	-	Hyperoside	Flavone	4.12E+06	5.33E+06	3.84E+06	4.25E+06	3.75E+06	4.25E+06	1.02E+07	7.12E+06	8.69E+06	1.57E+06	1.81E+06	2.60E+06	6.84E+06	4.98E+06	6.16E+06
pmf0208	4.65E+02	3.03E+02	3.90E+00	4.64E+02	[M+H]+	-	Isoquercitroside	Flavone	3.90E+05	4.85E+05	2.18E+05	5.09E+05	3.96E+05	9.00E+00	8.11E+05	5.67E+05	1.12E+06	9.00E+00	9.00E+00	1.85E+05	6.70E+05	4.01E+05	5.49E+05
pmf0216	4.43E+02	2.73E+02	3.94E+00	4.42E+02	[M+H]+	-	Catechin-gallate，CG	Polyphenol	6.04E+04	7.77E+04	4.84E+04	8.44E+04	4.73E+04	7.33E+04	3.86E+04	8.97E+04	8.79E+04	3.50E+04	3.16E+04	3.21E+04	5.41E+04	2.38E+04	4.31E+04
pmf0232	5.93E+02	2.85E+02	4.92E+00	5.94E+02	[M-H]-	C17140	Tiliroside	Flavone	3.91E+05	5.55E+05	5.66E+05	2.44E+05	1.88E+05	2.88E+05	4.78E+05	3.59E+05	3.88E+05	8.00E+05	1.01E+06	1.00E+06	9.00E+00	5.57E+04	9.00E+00
pmf0236	5.63E+02	2.41E+02	4.68E+00	5.64E+02	[M-H]-	-	Theaflavin	Polyphenol	3.29E+04	4.33E+04	4.69E+04	1.26E+04	1.01E+04	2.28E+04	1.99E+04	1.81E+04	1.41E+04	5.15E+03	1.40E+04	8.07E+03	2.14E+04	1.06E+05	5.50E+04
pmf0274	3.01E+02	2.11E+02	5.13E+00	3.02E+02	[M-H]-	-	Herbacetin	Flavone	9.27E+03	1.41E+04	1.47E+04	6.17E+03	2.34E+03	6.87E+03	1.55E+04	1.18E+04	1.43E+04	9.00E+00	9.00E+00	9.00E+00	9.00E+00	9.00E+00	9.00E+00
pmf0277	5.19E+02	5.01E+02	1.08E+01	5.18E+02	[M+H]+	C07667	Gossypol	Polyphenol	5.61E+04	7.58E+04	5.29E+04	5.31E+04	4.72E+04	7.15E+04	8.62E+04	7.05E+04	6.00E+04	1.41E+05	8.46E+04	7.43E+04	3.99E+04	9.00E+00	4.53E+04
pmf0278	4.65E+02	3.01E+02	4.00E+00	4.64E+02	[M-H]-	-	Gossypitrin	Flavone	2.09E+06	2.27E+06	2.76E+06	1.96E+06	1.93E+06	1.53E+06	4.09E+06	2.58E+06	2.91E+06	1.61E+06	1.93E+06	2.42E+06	2.28E+06	2.16E+06	2.60E+06
pmf0301	4.33E+02	2.69E+02	4.30E+00	4.34E+02	[M-H]-	-	Engeletin	Flavone	6.75E+04	8.43E+04	1.08E+05	9.00E+00	9.00E+00	9.00E+00	9.00E+00	9.00E+00	9.00E+00	2.84E+05	3.66E+05	3.71E+05	9.00E+00	9.00E+00	9.00E+00
pmf0345	2.75E+02	1.07E+02	3.74E+00	2.74E+02	[M+H]+	C12128	(-)-Epiafzelechin	Polyphenol	1.99E+05	1.92E+05	2.13E+05	6.26E+04	4.92E+04	7.96E+04	6.80E+04	9.53E+04	7.89E+04	1.67E+05	1.48E+05	9.78E+04	4.32E+05	4.53E+05	5.15E+05
pmf0361	4.51E+02	7.10E+01	3.99E+00	4.50E+02	[M+H]+	C17449	Astilbin	Flavone	3.29E+04	3.39E+04	2.64E+04	8.02E+03	9.00E+00	9.00E+00	9.00E+00	9.00E+00	9.00E+00	5.80E+04	8.84E+04	8.51E+04	9.00E+00	9.00E+00	9.00E+00
pmf0362	2.99E+02	2.27E+02	6.28E+00	3.00E+02	[M-H]-	-	Hydroxygenkwanin	Flavone	5.66E+03	7.08E+03	5.54E+03	9.00E+00	9.00E+00	9.00E+00	9.00E+00	5.51E+03	9.00E+00	9.00E+00	9.00E+00	9.00E+00	1.03E+04	1.10E+04	2.08E+04
pmf0371	3.15E+02	3.00E+02	4.72E+00	3.16E+02	[M-H]-	C10119	Pedalitin	Flavone	1.77E+04	3.89E+04	2.00E+04	1.02E+04	1.30E+04	2.37E+04	2.11E+04	3.66E+04	4.19E+04	9.00E+00	9.00E+00	9.00E+00	2.45E+04	1.25E+04	2.10E+04
pmf0375	4.79E+02	3.17E+02	4.18E+00	4.78E+02	[M+H]+	-	Isorhamnetin-3-O-glucoside	Flavone	4.44E+06	5.82E+06	5.13E+06	4.02E+06	3.86E+06	4.17E+06	1.91E+07	1.04E+07	1.10E+07	2.80E+06	3.18E+06	3.68E+06	5.62E+06	3.42E+06	5.56E+06
pmf0380	4.59E+02	1.39E+02	3.45E+00	4.58E+02	[M+H]+	-	Gallocatechin-gallate	Polyphenol	9.84E+04	9.83E+04	8.56E+04	1.21E+05	9.45E+04	1.47E+05	1.23E+05	1.93E+05	1.42E+05	8.22E+04	4.80E+04	1.02E+05	5.50E+04	3.14E+04	5.15E+04
pmf0417	5.95E+02	2.87E+02	3.70E+00	5.96E+02	[M-H]-	C09732	Eriocitrin	Flavone	1.54E+05	1.49E+05	1.42E+05	1.46E+05	1.44E+05	2.03E+05	1.46E+05	8.04E+04	1.20E+05	3.25E+05	3.18E+05	2.93E+05	9.00E+00	9.00E+00	9.00E+00
pmf0458	2.95E+02	2.77E+02	7.08E+00	2.94E+02	[M+H]+	C10462	6-Gingerol	Polyphenol	4.62E+04	7.37E+04	6.62E+04	2.67E+04	1.85E+04	2.79E+04	9.56E+04	1.28E+05	8.21E+04	1.14E+05	1.69E+05	1.51E+05	9.00E+00	9.00E+00	1.95E+04
pmf0492	4.15E+02	2.67E+02	3.12E+00	4.16E+02	[M-H]-	C10524	Puararin	Flavone	7.64E+02	6.31E+02	5.45E+02	3.60E+02	8.42E+02	6.52E+02	1.41E+03	1.78E+03	1.49E+03	1.78E+03	3.60E+02	7.38E+02	4.70E+02	1.31E+03	8.67E+02
pmf0521	3.03E+02	2.29E+02	5.13E+00	3.02E+02	[M+H]+	C10105	Morin	Flavone	8.56E+04	1.40E+05	1.74E+05	8.44E+04	8.46E+04	1.52E+05	2.13E+05	2.12E+05	2.36E+05	1.49E+05	9.00E+00	9.00E+00	9.00E+00	9.00E+00	9.00E+00
pmf0549	6.07E+02	2.99E+02	4.14E+00	6.08E+02	[M-H]-	C10039	Diosmin	Flavone	1.38E+07	1.32E+07	1.54E+07	9.27E+06	8.30E+06	1.02E+07	1.14E+07	1.19E+07	1.01E+07	2.22E+07	2.39E+07	2.14E+07	4.43E+05	5.86E+05	5.30E+05
pmf0569	4.17E+02	2.55E+02	3.87E+00	4.18E+02	[M-H]-	C16978	Liquiritin	Flavone	4.62E+04	4.84E+04	6.61E+04	2.17E+04	2.75E+04	4.20E+04	8.32E+04	5.40E+04	4.58E+04	3.21E+04	3.09E+04	3.62E+04	3.75E+05	1.64E+05	3.52E+05
pmf0584	4.37E+02	2.75E+02	4.37E+00	4.36E+02	[M+H]+	C01604	Phloridzin	Flavone	3.65E+05	3.64E+05	3.18E+05	1.07E+05	1.34E+05	1.54E+05	1.39E+05	1.96E+05	2.09E+05	2.06E+05	2.09E+05	2.44E+05	1.56E+06	2.06E+06	1.25E+06
pmf0614	6.25E+02	3.01E+02	2.00E+00	6.25E+02	Protonated	-	"Peonidin-3,-5-diglucoside-chloride"	Anthocyanins	1.87E+06	2.06E+06	2.06E+06	1.48E+06	1.19E+06	2.07E+06	1.62E+06	2.33E+06	1.88E+06	8.73E+06	8.47E+06	7.11E+06	9.00E+00	9.00E+00	9.00E+00
pmf0615	6.41E+02	3.17E+02	2.08E+00	6.41E+02	Protonated	-	"Petunidin-3,-5-diglucoside"	Anthocyanins	6.07E+07	7.47E+07	7.37E+07	1.96E+07	2.08E+07	2.49E+07	3.60E+07	2.15E+07	2.25E+07	9.00E+00	9.00E+00	9.00E+00	1.06E+08	1.13E+08	1.30E+08
pmf0616	6.97E+02	5.35E+02	3.03E+00	6.97E+02	Protonated	-	Malvidin-3-acetyl-5-diglucoside	Anthocyanins	4.50E+07	4.60E+07	3.86E+07	4.67E+07	4.64E+07	5.23E+07	4.67E+07	4.21E+07	3.68E+07	9.00E+00	9.00E+00	9.00E+00	2.61E+08	1.98E+08	2.45E+08
