# Supplementary figures and images for: The Metabolomic Profiling of the Flavonoid Compounds in Red Wine Grapes and the Impact of Training Systems in the Southern Subtropical Region of China
Source: Int J Mol Sci. 2024 Aug 7;25(16):8624. doi: 10.3390/ijms25168624 (PMC11354489; doi:10.3390/ijms25168624)

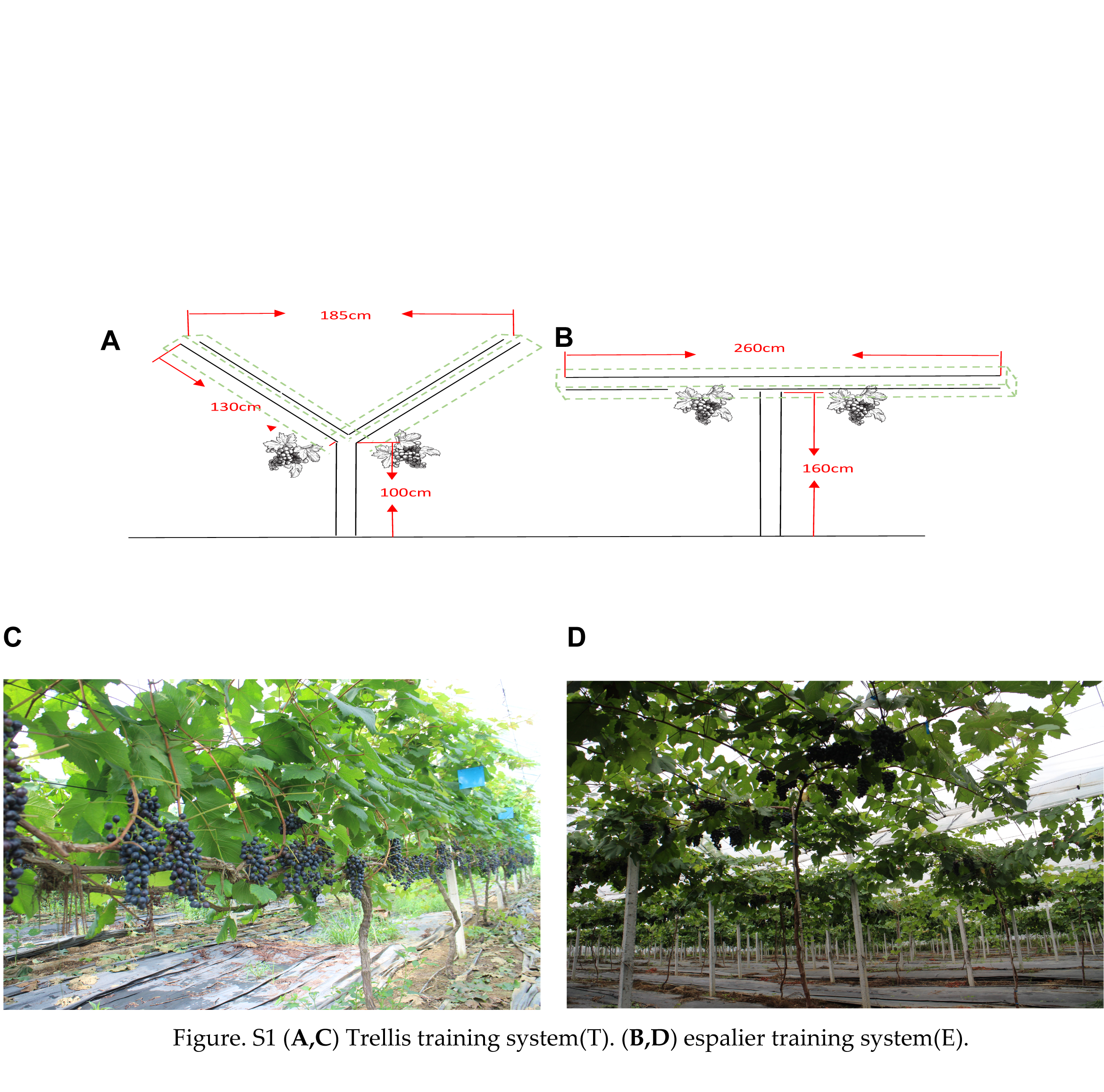

Supplement: Supplementary file 1 [file ijms-25-08624-s001.zip › ijms-3128253-Figure S1.tif]
